# Epigenetic Regulation of Processes Related to High Level of Fibroblast Growth Factor 21 in Obese Subjects

**DOI:** 10.3390/genes12020307

**Published:** 2021-02-21

**Authors:** Teresa Płatek, Anna Polus, Joanna Góralska, Urszula Raźny, Agnieszka Dziewońska, Agnieszka Micek, Aldona Dembińska-Kieć, Bogdan Solnica, Małgorzata Malczewska-Malec

**Affiliations:** 1 Department of Clinical Biochemistry, Jagiellonian University Medical College, 15a Kopernika Street, 31-501 Krakow, Poland; apolus@cm-uj.krakow.pl (A.P.); jgoralska@cm-uj.krakow.pl (J.G.); urazny@cm-uj.krakow.pl (U.R.); a.sliwa@uj.edu.pl (A.D.); mbkiec@cyf-kr.edu.pl (A.D.-K.); mbsolnic@cyf-kr.edu.pl (B.S.); mbmalec@cyf-kr.edu.pl (M.M.-M.); 2 Department of Nursing Management and Epidemiology Nursing, Faculty of Health Sciences, Jagiellonian University Medical College, 25 Kopernika Street, 31-501 Krakow, Poland; agnieszka.micek@uj.edu.pl

**Keywords:** FGF21, obesity, glucose and lipid metabolism, DNA methylation, microRNA

## Abstract

We hypothesised that epigenetics may play an important role in mediating fibroblast growth factor 21 (FGF21) resistance in obesity. We aimed to evaluate DNA methylation changes and miRNA pattern in obese subjects associated with high serum FGF21 levels. The study included 136 participants with BMI 27–45 kg/m^2^. Fasting FGF21, glucose, insulin, GIP, lipids, adipokines, miokines and cytokines were measured and compared in high serum FGF21 (*n* = 68) group to low FGF21 (*n* = 68) group. Human DNA Methylation Microarrays were analysed in leukocytes from each group (*n* = 16). Expression of miRNAs was evaluated using quantitative PCR-TLDA. The study identified differentially methylated genes in pathways related to glucose transport, insulin secretion and signalling, lipid transport and cellular metabolism, response to nutrient levels, thermogenesis, browning of adipose tissue and bone mineralisation. Additionally, it detected transcription factor genes regulating FGF21 and fibroblast growth factor receptor and vascular endothelial growth factor receptor pathways regulation. Increased expression of hsa-miR-875-5p and decreased expression of hsa-miR-133a-3p, hsa-miR-185-5p and hsa-miR-200c-3p were found in the group with high serum FGF21. These changes were associated with high FGF21, VEGF and low adiponectin serum levels. Our results point to a significant role of the epigenetic regulation of genes involved in metabolic pathways related to FGF21 action.

## 1. Introduction

Fibroblast growth factor 21 (FGF21) acts as an endocrine factor that regulates glucose and lipids homeostasis in humans and animals although its function is still debated due to its different sites of production and actions [1,2,3]. FGF21 is primarily expressed in the liver but also in the skeletal muscles, adipose tissue, gut and pancreas [4].

FGF21 is regulated by nutritional status (fasting state, high carbohydrate and high protein ingestion) as well as physical activity [3,5,6,7]. In humans, FGF21 expression in the liver was also found to be up-regulated by glucagon and by glucocorticoids, and repressed by insulin [3]. Circulating FGF21 concentrations exhibit a characteristic diurnal rhythm in humans [8]. Peroxisome proliferator-activated receptor α (PPARα) induces FGF21 transcription in the liver in the fasting state in humans and mice via binding to its promoter [3,9]. Nevertheless, other nuclear receptors such as retinoic acid receptor-related orphan receptor α (RORα), carbohydrate response element-binding protein (ChREBP), farnesoid X receptor (FXR) and liver X receptor (LXR) are also involved in the regulation of hepatic FGF21 expression [10,11,12]. Recently, the stimulatory effect of the fasting state (through KLF15 and BTG2) on hepatic FGF21 expression have been demonstrated in mice [13]. In contrast to the liver, FGF21 expression in brown and white adipose tissues (WAT) is induced mainly by cold exposure and adrenergic signalling as well as PPARγ, which directly mediates the expression of FGF21 in WAT [3,9]. In muscles, FGF21 expression is controlled by ATF4 [14].

FGF21 signalling to target cells is mediated by binding to and activation of four receptors: FGFR1, FGFR2, FGFR3 and FGFR4 in the presence of co-receptor β-klotho [15]. FGFR receptors are ubiquitously expressed, whereas β-klotho, an essential co-receptor, is expressed only in the liver, adipose tissue, pancreas and brain, suggesting that FGF21 functions selectively in these tissues [4]. Although therapeutic administrating of FGF21 prevents diet-induced obesity and related insulin resistance in mice and humans. Paradoxically metabolic disorders like obesity, diabetes are characterised by elevated serum FGF21. This highlights the important role of receptors. A recent study has shown the reduced expression of *FGFR1c*, *FGFR3c* and *FGFR4* receptors in skeletal muscles and increased expression of *FGFR1c* in WAT of individuals with type 2 diabetes [16]. Signal activation via FGFR1 leads to weight loss, brown fat thermogenesis, insulin sensitisation and hepatic lipids reduction [17]. FGF21 is widely known to exert multiple metabolic effects: increases insulin sensitivity, fatty acid oxidation and ketogenesis but inhibits lipogenesis in liver; increases fatty acid uptake, lipolysis, browning, thermogenesis and mitochondrial activity in white adipocytes and enhances glucose uptake and thermogenesis in brown adipocytes [3,12].

Serum level of circulating FGF21 increases with age as well as in obesity and insulin resistance state [18,19,20]. High serum FGF21 level is associated with MAFLD (metabolic associated fatty liver disease) and the higher risk of developing metabolic syndrome and type 2 diabetes [21,22]. Elevated FGF21 levels are observed in patients with atherosclerosis, pancreatitis and reduced bone strength [3]. Moreover, recent findings have indicated that high versus low serum FGF21 levels correlate with mortality in the elderly [12]. FGF21 is a part of a complicated inter-organ cross talk, so other factors (e.g., cytokines, metabolites) may modulate FGF21 action in target tissues and consequently elicit a beneficial (healthy) or detrimental (unhealthy) effect.

It is hypothesised that proper action of FGF21 prevents the development of diet-induced obesity [21]. According to a recent hypothesis, FGF21 is a hormonal mediator of the human “thrifty” metabolic phenotype [23]. Efficient regulation, with large changes in FGF21 secretion in response to the supply of nutrients or nutrient deprivation, is one of the main factors determining the metabolic phenotype less susceptible to the development of obesity and its complications [24]. As FGF21 also plays a role in brown adipose tissue activation, it seems, to some extent, to affect the individual propensity to weight gain. However, in obesity the state of FGF21 resistance is widely observed, which is suggested as compensatory up-regulation response to chronically elevated serum levels of FGF21 [19]. Mechanisms mediating the interaction between environmental factors and the genome, such as epigenetics, may be of particular importance in the pathogenesis of FGF21-resistance. In recent study on mice it was demonstrated that Fgf21 gene methylation status can be modulated in a PPARα-dependent manner [25]. Therefore, expanding knowledge of the epigenetic basis associated with blunted FGF21 response to diet, is important for identifying individuals at risk and preventing the metabolic complications of obesity.

The aim of this study was to find differentially methylated genes and profile of miRNAs related to high FGF21 levels in obese subjects. For this purpose, a genome wide DNA methylation and miRNA expression patterns were determined in leukocytes. We chose blood samples, as easily accessible to collect, for screening of epigenetic biomarkers associated with high FGF21 levels. As a result, a blood epigenetic profile characteristic for high serum FGF21 in obesity was established.

## 2. Materials and Methods

### 2.1. Patients

The study group (from the BIOCLAIMS cohort) included adult overweight and obese individuals (*n* = 136; 100 women and 36 men) with BMI ranging from 27 to 45 (overweight: *n* = 30, obese: *n* = 106). Exclusion criteria were cardiovascular diseases, diabetes mellitus, kidney or liver failure, endocrine disorders and chronic inflammation as well as smoking or excessive use of alcohol, pregnancy or lactation. Subjects did not use any drugs or dietary supplements and led inactive lifestyle.

The study was conducted in accordance with the Code of Ethics of the World Medical Association (Declaration of Helsinki) and with the Good Clinical Practice guidelines. The study protocol was approved by the Bioethics Committee of the Jagiellonian University, Krakow, Poland (file number: KBET/82/B/2009 and KBET/45/B/2012).

Patients were divided into 2 groups based on the fasting serum FGF21 levels (FGF21 < 213 pg/mL and ≥ 213 pg/mL). The cut-off point was the median serum FGF21 in the cohort.

### 2.2. Anthropometry Measurements

All subjects had measurements of body weight, height, waist and hip circumferences and blood pressure. Body fat percentage was estimated by bioelectrical impedance method using the Segmental Body Composition 116 Analyzer TANITA BC 418 MA (Tanita Corporation, Tokyo, Japan).

### 2.3. Sample Collection and Biochemical Tests

Fasting venous blood samples were always between 8 to 9 am collected and then centrifuged (1000 × g for 10 min at 4 °C) within 30 min of collection for serum and plasma separation. For biochemical tests (except free fatty acids (NEFAs), serum and plasma samples were immediately frozen and stored at −80 °C until assayed. For analysis of DNA methylation, fasting venous blood was collected into K3-EDTA tubes. At the same time, a second sample of venous blood was collected into PAX gene Blood RNA Tubes (Becton Dickinson, Bedford, MA, USA) for miRNAs expression analysis. All samples were frozen and stored at −80 °C until analysed.

Serum was used for biochemical tests: glucose, insulin, non-esterified fatty acids (NEFA), total cholesterol, HDL-cholesterol and triglycerides (TG) concentrations. Glucose, total cholesterol, HDL cholesterol, and TGs were assayed using enzymatic colorimetric methods on the MaxMat analyzer (MaxMat S.A., Montpeliere, France). LDL cholesterol concentration was calculated using the Friedewald formula. Insulin was measured by an immunoradiometric method (INS IRMA Diasource ImmunoAssays, Neuve Belgium). Homeostasis model of assessment (HOMA-IR) was calculated as a ratio according to Matsuda M and DeFronzo RA [26]. Plasma GIP was measured by ELISA (Human GIP (Total) 96-Well Plate Assay (EMD Millipore, St Charles, MO, USA)). Total plasma fatty acids content and composition of saturated, monounsaturated and polyunsaturated fatty acids were measured by gas-liquid chromatography and flame-ionization detector after direct in situ transesterification, according to Glaser et al. [27]. NEFAs concentrations were measured immediately in fresh plasma by an enzymatic quantitative colorimetric method (Roche Diagnostics GmbH, Mannheim, Germany). Activities of ALT and GGT were measured by the routine method using an automated analyser (Hitachi cobas c 701/702, Roche Diagnostics GmbH, Mannheim, Germany).

### 2.4. Adipokines, Miokines and Cytokines

Serum concentrations of sEselectin, monocyte chemoattractant protein 1 (MCP-1), vascular cell adhesion protein 1 (sVCAM-1) and interleukin-6 (IL-6) were measured using ELISA kits (Human sE-Selectin/CD62E Quantikine ELISA, Human CCL2/ MCP-1 Quantikine ELISA, Human sVCAM-1/CD106 Quantikine ELISA and Human IL-6 Quantikine HS ELISA Kit, R&D Systems, USA). Soluble platelet/endothelial cell adhesion molecule 1 (sPECAM-1) was measured by ELISA kit (BioVendor, Brno, Czech Republic) and hsCRP was measured by immunoturbidimetric method (APTEC Diagnostics nv, Sint-Niklaas, Belgium). Adiponectin and leptin were measured using R&D Systems kits (Human Total Adiponectin/Acrp30 Quantikine ELISA kit and Human Leptin Quantikine ELISA kit; R&D Systems Inc. Minneapolis, MN, USA). For the quantitative determination of human visfatin the Human Visfatin (Nampt/ PBEF) ELISA kit (BioVendor—Laboratorni Medicina a.s. Brno, Czech Republic) and for human resistin (Human Resistin Quantikine ELISA Kit (R&D Systems Inc. Minneapolis, MN, USA) were used. To measure serum FGF-21 and Vascular endothelial growth factor (VEGF) concentrations, the ELISA kits (Human FGF-21 Quantikine ELISA Kit and Human VEGF Quantikine ELISA Kit, R&D Systems Inc. Minneapolis, MN, USA) were used. Human myostatin was assayed using the Human Myostatin ELISA kit (Immunodiagnostik AG, Bensheim, Germany).

### 2.5. Genome-wide DNA Methylation Analysis

DNA methylation microarrays were analyzed in 16 samples selected randomly (matched to sex and BMI) from the two studied groups (8 representative for each group- High FGF21 and Low FGF21). The subgroups reflected the biochemical characteristics of the group they represented. Genomic DNA was isolated from peripheral blood leukocytes using the High Pure PCR Template Preparation Kit (Roche Diagnostics, Mannheim, Germany). The assessment of DNA quantity and quality was performed by spectrophotometry using the NanoDrop ND1000 UV-VIS spectrophotometer (Thermo Fisher Scientific, Waltham, MA, USA). First, sonication of 5 μg of DNA was performed. Sonication efficiency was assessed by electrophoresis on a 2.0% agarose gel. The 4/5 of sonicated DNA was taken for immunoprecipitation, the fifth part was used as a reference input fraction. The immunoprecipitation of DNA containing 5-methylcytosines (5-mC) were performed using monoclonal antibodies against 5-methylcytidine (Monoclonal Antibody to 5-Methyl Cytosine / 5-MeC Purified from Acris Antibodies, Inc, USA). Immunoprecipitated and reference DNA aliquots were labelled with fluorescent dyes Cyanine-3 and Cyanine-5, respectively. Competitive hybridisation of input material and methylated DNA enriched fraction was performed to oligonucleotide microarrays—Human DNA Methylation Microarray: High-definition 244K arrays contained 27,627 probes for annotated human CpG islands and 5081 undermethylated regions (G4495A, Design ID: 023795) from Agilent Technologies (Santa Clara, CA, USA). The selected arrays includes 3 types of probes: probes for known CpG islands, probes spanning 85 nucleotides proximal to each CpG island and probes for undermethylated regions representing 237,227 unique biological probes. Microarrays were hybridized for 40 h at 65 °C. Samples amplification and labelling, hybridisation, slides washing and scanning procedures and image extraction using Agilent Features Extraction software v 10.10.1.1 were performed according to the manufacturer’s instructions.

### 2.6. Determination of miRNAs Expression by Real-Time PCR -TLDA (TaqMan Low Density Arrays)

For relative quantification of miRNA, 16 blood samples intended for epigenetic analysis were drawn from both group (8 representative for each group- High FGF21 and Low FGF21).

MiRNA was extracted using the PAXgene 96 Blood RNA Kit and GeneMATRIX Universal RNA/miRNA Purification Kit (EUR_X_, Gdansk, Poland). Finally, after isolation we obtained miRNAs from peripheral blood leukocytes. MiRNA quality was assessed in an Agilent Bioanalyzer 2100 using the RNA 6000 Nano kit (Agilent Technologies, Santa Clara, CA, USA) and quantified by spectrophotometry using the NanoDrop ND-1000. For miRNAs expression analysis by Real-time PCR, TaqMan Low-Density Array (TLDA) (TaqMan^®^ Array Human MicroRNA A+B Cards Set v3.0, Thermo Scientific, Wilmington, DE, USA) was used. Selected arrays allow simultaneous and accurate quantitation of 754 human microRNAs. Reverse transcription was performed with TaqMan MicroRNA Reverse Transcription Kit and MegaPlex Human Pool A and B and RT primers. The preamplification step was enabled by Megaplex™ PreAmp Kit and Primers. The arrays were run in a 7900HT Fast Real-Time PCR system (Thermo Scientific, Wilmington, DE, USA).

### 2.7. Real-time PCR Analysis

Total RNA was reverse transcribed using a reverse transcription kit (High Capacity cDNA Reverse Transcription Kit (Applied Biosystems, Carlsbad, CA, USA)) with random primers (1 µg). Quantitave real-time polymerase chain reaction (qPCR) was performed with the QuantiTect SYBR Green PCR kit. The specific TaqMan Gene Expression Assays with a pair of specific PCR primers and a TaqMan probe with a FAM (Applied Biosystem, Carlsbad, CA, USA) was used. Amplification was performed using the continuous fluorescence detection system 7900 HT Fast Real Time PCR system (Applied Biosystem, Carlsbad, CA, USA). The expression ratio of target mRNA was normalised to the level of 18 s RNA and compared to control group. Data was analysed using the ΔΔCT method. The statistically significant results were recognized, with a value of *p* < 0.05.

### 2.8. Statistical Analyses

For biochemical and anthropometric parameters, data distribution was analysed by Shapiro-Wilk test. Differences between groups were calculated by unpaired t-test for normally distributed data and Mann–Whitney U-test for non-normally distributed data (BMI, adipose tissue mass, WHR, glucose, HOMA-IR, VEGF, GIP, leptin, adiponectin, FGF21, myostatin, IL-6 and hs-CRP). The chi-squared test was used for nominal variables. Normally distributed data are presented as mean ± standard deviation (SD), otherwise as median (Q2) and interquartile range (Q1; Q3). All analyses were performed with the Statistica 13 software (StatSoft Polska, Krakow, Poland). The *p*-value < 0.05 was considered statistically significant.

Microarray DNA methylation data analysis was performed using the Feature extraction software version 10.10.1.1 (Agilent Technologies, Santa Clara, CA, USA), the BRB-ArrayTools software version 4.6 (National Institutes of Health, Bethesda, MD, USA) and the R software version 3.6.1 (Development Core Team, Vienna, Austria and Bioconductor). Feature extraction software was used to assess background subtracted intensity values for the two fluorescence dyes on each individual array feature and calculated as the ratio (Cy3/Cy5). Loess normalisation method for dual channel raw hybridisation signals were computed in BRB-ArrayTools. M values (log intensity ratio M = log_2_ (Cy3/Cy5)) were used for downstream statistical analyses. A linear regression model for comparing two groups with high and low FG21 was adjusted for potential confounders including blood cell-type proportions and age. To account for potential differences in the proportions of blood cells, we estimated the proportion of lymphocytes, monocytes, eosinophils, basophils and neutrophils to adjust raw data as previously described by Houseman et al. in R [28]. We used the functions lmFit and eBayes in “limma” package to build linear models and compute moderated t-statistics (p-value was calculated). Then Benjamini–Hochberg multiple test correction FDR was performed and shown as q-value. Regarding methylation sites, probes not including CpG or not located in CpG islands, probes containing CNVs in the CpG site and probes located on X and Y chromosomes were removed from the analyses. Fold of change (FC) in relation to obese with high FGF21 versus obese with low FGF21 was used as the methylation level. Loci corresponding to q-value < 0.01 and FC in the range of > 1.4 or < −1.4 were classified as differentially methylated. Methylated regions had log ratios significantly above zero while less methylated regions had log ratios significantly below zero. Highly methylated regions are with FC ≥ 2.0 and moderately methylated with FC between 1.4 to 2.0.

MiRNAs TLDA arrays data analysis was calculated using DataAssist v 3.01 tool (Thermo Scientific). Relative miRNA levels were expressed as fold of change ((FC) = geometric mean 2(−∆Ct high FGF21) / geometric mean 2(−∆Ct low FGF21)), in the presence of 3 genes (RNU44, RNU48, U6sRNA) used as endogenous controls. MiRNAs with fold of change (FC) ≥ 2.0 were taken for further analysis.

Pathway analyses:

We used the BiNGO plugin in Cytoscape software (version 3.7.2) to assess the involvement of selected genes in biological processes and molecular function [29]. The parameters as overrepresentation after correction (using statistical test as hypergeometric test with multiple testing correction as Benjamini–Hochberg False Discovery Rate (FDR) correction) were selected. Results with corrected *p*-value < 0.05 are presented in this manuscript.

The putative target predictions of miRNAs were extracted using the three online software solutions: mirPath v.3 DIANA TOOLS, miRTarBase and miRWalk databases [30,31,32].

## 3. Results

### 3.1. Biochemical and Anthropometric Characteristics of the Study Groups

The two investigated groups were comparable in terms of age, sex, BMI, adipose tissue mass, blood pressure, fasting glucose and cholesterol, regardless of serum FGF21 levels. Nevertheless, the group with high circulating FGF21 was characterised by higher WHR, elevated fasting insulin and HOMA-IR, NEFAs as well as triglycerides levels (Table 1). This group included 42 (62%) individuals who met the criteria for abdominal obesity, compared to the low FGF21 group, *n* = 27 (40%). Plasma GIP, an incretin hormone important for regulating carbohydrate as well as lipid metabolism, was also found to be elevated in the high FGF21 group. Liver damage markers (ALT, GGT) also differed between groups, indicating patients with high FGF21 are more prone to liver injury. Obese patients with high FGF21 had elevated VEGF, MCP1 and decreased adiponectin levels whereas other adipokines—leptin, resistin, visfatin, myokines—irisin, myostatin, and IL-6 levels did not differ between these groups (Table 1). Other inflammatory markers studied—CRP, sVCAM, sPECAM, sE-selectin—showed comparable levels regardless of circulating FGF21 (Table 1). The patients in the study group presented metabolic profile related to elevated FGF21. Characteristics of subjects selected for epigenetic study are presented in Table S1 (in supplementary files).

### 3.2. Epigenetics Study Results

#### 3.2.1. Results of DNA Methylation Analysis

The analysis of the data obtained from DNA methylation arrays revealed 11 198 CpG probes differentially methylated (*p* < 0.01, representing approximately 4.6% of probes located on the array), of which 5425 were significantly hypomethylated and 5774 were significantly hypermethylated in the high FGF21 group compared to the low FGF21 group. We selected 375 CpG probes for further analysis of biological significance in pathways related to potential FGF21 action. We focused on those genes that were engaged in metabolism of lipids, glucose and adipokines and other pathways known or potentially related to FGF21 activity. Detailed results of each probe described in the manuscript are presented in Table S2 enclosed as a supplementary file.

Analyses in Cytoscape software revealed biological processes affected by differentially methylated genes (results presented in Table 2). The most interesting finding is the involvement of these genes in the response to glucose and insulin and other nutrient levels. Subsequently, the differential methylation status of lipid transport and fatty acid metabolism, fat cell differentiation (including white and brown fat cell differentiation), generation of precursor metabolites and energy, response to hypoxia and regulation of ossification, were demonstrated (Table 2). Importantly, in the high FGF21 group, changes in methylation status of genes involved in browning program of adipose tissue: *BMP4* (hypomethylated in promoter), *FGF9* (hypermethylated in promoter) as well as *PRDM16* (hypermethylated in promoter) and *CPT1C* (hypomethylated in promoter) were detected. Genes: *MRAP* (shown as hypomethylated in the high FGF21 group) and *TBX1* (hypermethylated in the high FGF21 group) are the hallmark proteins, for fatty tissue (highly enriched in differentiated adipocytes) and beige adipocytes, respectively.

Interestingly, five genes coding for transcriptional factors: *PPARA*, *RORA*, *ATF4*, *NR3C1* and *KLF15*, known to regulate FGF21 expression, were differentially methylated (Table 3). Analysis of DNA methylation also revealed regulation of fibroblast growth factor receptor signalling (hypomethylated genes: *FGF3*, *KLB*, *FGFR1* and *FGFR3*) and vascular endothelial growth factor receptor signalling (hypomethylated genes: *VEGFA* and *FLT1*) pathways (Table 2). High FGF21 individuals were also characterised by the *LIPG* gene, as well as *ABCA1* and *VLDLR* genes (all hypermethylated) related to lipoprotein metabolism and transport. We found differential methylation in promoters in genes: *IGF1R* -receptor for IGF1 and *IGFBP1-* an FGF21-induced pro-osteoclastogenic liver hormone (all with hypermethylated status in promoters in the high FGF21 group) (Table 2). Interestingly, the promoter of *GIPR* (receptor for GIP) gene was hypermethylated in this group. We showed at the protein level that serum GIP and VEGF levels were significantly higher in obese patients with high FGF21 than in subjects with low FGF21.

Further analysis of differentially methylated genes in ClueGO plugin in CytoScape software allowed to create graphic representation of biological processes and pathways (Figure S1). Figure S1 is enclosed as a supplementary file.

DNA microarray results was verified by real-time PCR in 17 samples (8 from high FGF21 group and 9 from low FGF21 group) for selected *ATF4* gene. We demonstrated a statistically significant 2.75-fold decrease in expression of the *ATF4* gene in leukocytes (*p* < 0.05). This result confirms that detected DNA hypermethylation in the promoter of ATF4 gene regulates its expression in patients with high serum FGF21 level.

#### 3.2.2. Results of microRNA Expression Level in Leukocytes:

We demonstrated a statistically significant difference in expression of four miRNAs in peripheral blood leukocytes related to high FGF21 serum levels. Expression of the following miRNAs: hsa-miR-133a-3p, hsa-miR-185-5p and hsa-miR-200c-3p was downregulated and hsa-miR-875-5p was up-regulated (results are presented in Table 4). In Table 4, we also selected targets for miRNAs in relation to high FGF21 (among others the FGFR1 gene, receptor for FGF21) as well as related to high serum VEGF (VEGFA and its binding receptors genes) and low adiponectin (the ADIPOQ gene), found to accompany high FGF21 serum levels. Presented in Table 4, target genes for hsa-miR-133a-3p, hsa-miR-185-5p and hsa-miR-200c-3p were validated in previous manuscripts and/ or information based on selected online datasets. For hsa-miR-875-5p, we chose some putative target genes located in 3’UTR from miRWalK and TargetScan databases.

## 4. Discussion

In the current study, we have demonstrated for the first time genome-wide DNA methylation profile and miRNAs expression analysis in white blood cells associated with high serum FGF21 levels in human obese non-diabetic individuals. In our cohort, well-characterised in terms of many circulating active mediators, such as myokines, adipokines, incretins, cytokines and fatty acids, we observed increased parameters of insulin- and GIP resistance, lipids disorders, elevated VEGF, MCP1 and lower adiponectin serum levels suggesting disturbed nutrients signalling and disregulated inter-organ cross-talk in the high FGF21 group compared to the low FGF21 group.

It is well known that the altered DNA methylation of CpG sites in the genes’ promoters, as well as distal regulatory sites, changes gene expression patterns by modifying the interaction of histones, thereby affecting the binding of transcription factors or recruitment of methyl-CpG binding proteins (MBPs) [39,40]. The detected and presented, differentially methylated genes may be responsible for aberrant expression of genes related to high FGF21 level in obese people.

As FGF21 is responsible mainly for glucose and lipid homeostasis we showed altered methylation in a set of genes involved in metabolic pathways of glucose transport (*SLC2A4, SLC2A5, SLC2A8* and *KLF15*), insulin secretion and signalling (*ADRA2A, CPT1A, GIPR, IGF1R, IGFBP1, INSR, IRS1, NEUROD1, PDK1, PFKM, PPARA, PRKCI, SLC27A1, SLC2A8, SOCS3, TCF7L2* and *VLDLR*) as well as lipid transport, fatty acid metabolism and lipoprotein metabolism and transport *(ABCA1, ABCG4, ACOX3, ACOXL, ACSL3, ADIPOR1, CPT1A, CPT1B, CPT1C, DECR1, ELOVL4, ELOVL6, ELOVL7, FADS2, LPIN1, PPARA, PRKAA1, PRKAB2, PRKAR2B, SCD* and *SLC27A1*). Interestingly, the gene encoding the receptor for insulin (*INSR*) was hypomethylated. Contrary to this, we found hypermethylation of the gene *SLC2A4* (coding for GLUT4, insulin-responsive glucose transporter type 4), which plays a role in glucose uptake from the circulation and has been determined a key regulator of whole body glucose homeostasis [41].

Regarding lipid metabolism, differentially methylated genes: *PPARA*, *LPIN1*, *CPT1A, CPT1B, CPT1C, ANGPTL4* and *FADS2* are key regulators of liver, adipose tissue, muscle and total lipid homeostatis. *PPARA* (nuclear transcription factor) together with *LPIN1* (acting as a nuclear transcriptional coactivator for PPARGC1A and PPARA) regulate various lipid metabolic pathways [42,43]. In the liver PPARA induces the expression of genes involved in fatty acid oxidation, binding, activation, elongation and desaturation, synthesis and hydrolysis of triglycerides and lipid droplets, lipoprotein metabolism, gluconeogenesis and bile acid metabolism pathways [44]. It has been demonstrated on mice models that enhanced lipin expression increases lipogenic gene expression in adipose tissue and promoted fat storage in obesity, whereas in skeletal muscle it participated in energy expenditure and fat utilisation [45]. *CPT1C* gene plays a role in the central control of feeding behaviour and whole-body energy homeostasis [46]. Hypomethylation of leukocytic *CPT1C* gene was previously thought to be associated with increased risk of developing MAFLD [47].

Among five detected TFs known to regulate FGF21 expression, the majority (except 1 probe in *NR3C1* gene) were hypermethylated in patients with high FGF21. *PPARA* was activated by endogenous fatty acids and other PPARα agonists and, in turn, activated expression of FGF21 and genes involved in fatty acids beta-oxidation and ketogenesis pathways [33,48]. Research on mice showed that PPARα modulated the methylation of liver Fgf21 and represents a form of epigenetic memory that persists from the postnatal period into adulthood that influence the risk of obesity in later life [25]. On the other hand, we did not observe any differences in methylation status of *PPARG*, regulating FGF21 expression in WAT, between the groups. It has been reported previously that glucocorticoid receptor decreases hepatic PPARα expression and, in consequence, reduces liver FGF21 expression and plasma levels [10] and suppression of nuclear receptor RORA causing a decrease in FGF21 expression [11]. Observed hypermethylation of TFs (*KLF15*, *NR3C1* (glucocorticoid receptor), *RORA* (all regulating hepatic FGF21 expression) and *ATF4* (regulating muscle FGF21 expression)) suggests disturbed regulation of FGF21 in tissues. It was identified that glucose induced Fgf21 mRNA expression through ChREBP activation, as the ChoRE is located in the Fgf21 promoter. The feedback systems of ChREBP regulation are composed of two factors: PPARα and Fibroblast growth factor-21. Negative feedback is achieved by FGF21, which interacts with PPARα to repress their own transcription [49]. Although PPARα is the most important regulator of hepatic FGF21 expression, it is possible that other TFs may be down-regulated by FGF21, which is suggested by our DNA methylation results.

In our study, besides adipomyokine FGF21, two other myokines-myostatin and irisin-were measured in the serum. Since circulating myostatin and irisin did not differ between the groups, we supposed that FGF21 was mainly of hepatic origin and not skeletal muscle. Thus, we postulated that high FGF21 levels in our studied group were associated with a metabolic disorder, not induced by exercise. Receptors mediate FGF21 action in target tissues. FGFR1 and FGFR2 are ubiquitously expressed growth factor receptors that mediate most biological functions of the FGF family [4]. Regarding liver FGF21 receptors, hepatic mRNA expression is the highest for FGFR4 and FGFR2, then for FGFR3 and the lowest for FGFR1 [50,51,52]. We detected differential methylation in *FGFR1* and *FGFR3* receptor genes and their co-factors: *FGFRL1* and *KLB* genes. This is in line with previous studies where it was demonstrated that hepatic levels for β-Klotho, FGFR1 and FGFR3 transcripts were significantly increased in patients with obesity [53]. Interestingly, patients in the high FGF21 group presented simultaneously high level of serum VEGF protein. We detected differential DNA methylation in pathway: positive regulation of vascular endothelial growth factor receptor signalling (with affected genes: *FLT1, VEGFA* and *FGF9*).

As non-coding microRNAs are post-transcriptional regulators of genes translation, we analysed the microRNA profile in patients with obesity and high FGF21 levels. Analysis of miRNAs expression in leukocytes revealed a statistically significant decrease in expression of has-miR-133a-3hashsa-miR-185-5hasnd hsa-miR-200c-3p in the group with high FGF21. Previously, it was demonstrated that miR-133 directly targeted and negatively regulated Prdm16 in mice [54]. In humans, it was shown that circulating miR-133 is high in patients with periprocedural myocardial injury and, as a consequence, decreased fibroblast growth factor (FGFR1) [55]. As FGF21 is known to be activated by stress conditions, low hsa-miR-133a-3p expression in our studied group may be a kind of response to metabolic stress. According to a recent hypothesis, FGF21 is a hormonal mediator of the human “thrifty” metabolic phenotype; thus, it may be one of the mechanisms of metabolism towards browning of adipose tissue. In studies on human cancer cells inverse correlation of miR-185 and miR-200c to modulate VEGFA translation was found [56,57]. This profile of miRNAs is in line with elevated serum VEGF level detected in the group with high FGF21 from our cohort. The present study revealed the increased expression of has-miR-875-5p. There is limited information regardihashsa-miR-875-5p. Potential involvemehasof hsa-miR-875-5p to regulate TNF, LEP and IRS1 genes in gestational diabetes mellitus was demonstrated in a recent study [58]. Previous studies have shown decreased lhasls of hsa-miR-875-5p in the adipose tissue of patients with non-alcoholic steatohepatitis (NASH) [59]. Searching the miRNAs databases, we found potential targets for hsa-miR-875-5p such as: *ADIPOQ* (Adiponectin), *BMP8A* (Bone Morphogenetic Protein 8a), *BMPR1A* (Bone Morphogenetic Protein Receptor Type 1A), *CPT1A* (Carnitine Palmitoyl transferase 1A), *IRS1* (Insulin Receptor Substrate 1), *LIPA* (Cholesteryl Esterase), *SLC2A2* (Glucose Transporter Type 2, Liver), *SLC2A3* (Glucose Transporter Type 3, Brain) and *SLC2A4* (Glucose Transporter Type 4, Insulin-Responsive) genes. Selected genes are the key genes regulating lipid and glucose metabolism and may be potential targets for FGF21 action on metabolism. Simultaneously, we demonstrated statistically significant lower adiponectin protein level in serum in individuals with high FGF21; however, to confirm that hsa-miR-875-5p regulate the ADIPOQ gene are required further miRNA/mRNA interaction validations.

The main limitation of our study was the analysis of genome-wide DNA methylation and miRNAs expression in limited samples representative for both groups. Thus, results from our high-throughput method gives rise for further studies on targeted DNA methylation. We assessed DNA methylation in peripheral blood cells because they are easily accessible and the collection is acceptable by patients. Although epigenetic studies on different tissue samples are more informative, blood samples are generally used in most studies with non-surgery subjects. Nevertheless, previous studies demonstrated that levels of DNA methylation in blood tend to be broadly correlated with levels in other target tissues [60,61]. This suggests that DNA methylation and miRNAs analysis in leukocytes may reflect epigenetic changes in other tissues (liver, muscles, adipose tissue and bones) relevant for the pathogenesis of glucose and lipid disturbances related to high FGF21 in obesity. Another limitation is investigations among participants with metabolically healthy obesity, excluding general obese population with associated diabetes and cardiovascular diseases or other obesity complications.

## 5. Conclusions

The major finding of this preliminary study is that individuals with high FGF21 presents specific DNA methylation and miRNAs profiles in blood leukocytes. Altered DNA methylation in promoters of transcription factor genes (*PPARA, KLF15, NR3C1, RORA* and *ATF4*) known to regulate FGF21 expression and its binding receptors and co-factors (*FGFR1, FGFR3* and *FGFRL1* and *KLB*) has been revealed in relation to the elevated levels of circulating FGF21 in obesity. The mostly regulated processes are insulin secretion and signalling, lipid transport and homeostaais maintenance, thermogenesis and browning of adipose tissue and regulation of ossification and bone mineralization. Additionally, we demonstrated differentially expressed miRNAs known to target and inversely regulate the *FGFR1* and *VEGFA* genes. As DNA methylation is reversible and depends on environmental factors, there is the potential to influence the methylation status of key genes by nutrition and a healthy lifestyle to prevent obesity-related complications [62,63,64,65]. These findings give rise for further studies on detailed dietary factors and targeted DNA methylation editing therapies that may regulate revealed genes.

## Figures and Tables

**Table 1:** Characteristics of subjects.

Race (Caucasian)	High FGF21 (*n* = 68)		Low FGF21 (*n* = 68)		*p*
**Sex. F/M (%)**	**63**		75		Ns
Age (years)	48.3	±11.5	47.2	±11.0	0.582
BMI (kg/m2)	32.90	(30.4–36.2)	32.3	(30.3–34.6)	0.235
Adipose tissue mass (%)	39.7	(33.7–42.7)	38.2	(34.1–42.9)	0.969
**WHR**	**0.92**	**(0.85–1.02)**	**0.84**	**(0.80–0.92)**	**0.001**
**Waist circumference (cm)**	**108.5**	**±12.1**	**100**	**±11.1**	**0.001**
Systolic blood pressure (mmHg)	130.6	±14.9	127.7	±15.9	0.246
Diastolic blood pressure (mmHg)	84.4	±8.6	83.2	±9.3	0.427
Fasting glucose (mmol/L)	5.4	(5.0–5.7)	5.2	(4.7–5.5)	0.193
**Insulin (µIU/mL)**	**18.1**	**±10.4**	**14.5**	**±7.7**	**0.035**
**HOMA-IR**	**3.29**	**(2.45–4.9)**	**2.71**	**(2.0–4.04)**	**0.038**
**VEGF (pg/mL)**	**361.1**	**(226.7–521.1)**	**263.2**	**(176.4–421)**	**0.038**
Total cholesterol (mmol/L)	5.7	±1.0	5.4	±1.1	0.061
HDL cholesterol (mmol/L)	1.3	±0.3	1.3	±0.2	0.236
LDL cholesterol (mmol/L)	3.6	±0.9	3.4	±1.0	0.192
**TG (mmol/L)**	**1.8**	**±0.9**	**1.3**	**±0.7**	**0.0004**
**GIP (pg/mL)**	**31.6**	**(18.8–45.6)**	**25.5**	**(16.1–33.1)**	**0.019**
**ALT (U/L)**	**20.8**	**±12.0**	**16.8**	**±8.3**	**0.026**
**GGT (U/L)**	**34.2**	**±39.3**	**19.8**	**±11.9**	**0.005**
Leptin (ng/mL)	36.1	(20.3–56.3)	27.5	(20.8–44.6)	0.155
**Adiponectin (µg/mL)**	**4.9**	**(3.8–7.55)**	**7.3**	**(5.3–9.7)**	**0.013**
Resitin (ng/mL)	9.5	±3.7	10.4	±3.9	0.120
Visfatin (ng/mL)	1.2	±0.9	1.1	±0.9	0.439
**FGF21 (pg/mL)**	**299**	**(253.8–401.9)**	**127.8**	**(62–162.4)**	**<0.0001**
Irisin (µg/mL)	5	±1.6	4.6	±1.2	0.112
Myostatin (ng/mL)	21.5	(18.5–24.3)	21.2	(18.7–25.05)	0.964
IL-6 (pg/mL)	1.3	(0.9–1.9)	1.2	(0.8–1.7)	0.411
hs-CRP (mg/mL)	2.2	(1.0–3.8)	1.1	(0.5–3.8)	0.191
**MCP1 (pg/mL)**	**392.1**	**±127.2**	**348.2**	**±102**	**0.034**
sVCAM-1 (ng/mL)	609	±160.1	636.9	±149.1	0.270
sPECAM-1 (ng/mL)	74.6	±19.9	71.7	±16.9	0.395
sEselectin (pg/mL)	43.8	±20.7	39.4	±15.6	0.179
**Fasting NEFAs (mmol/L)**	**0.804**	**±0.314**	**0.674**	**±0.268**	**0.008**
**Total fatty acids (µg/mL)**	**3642.9**	**±819.8**	**3258.9**	**±737.1**	**0.005**
Saturated fatty acids (%)	33.4	±2.6	33	±1.9	0.351
Monounsaturated fatty acids (%)	26.3	±2.7	25.4	±3.0	0.051
Polyunsaturated fatty acids (%)	40.3	± 4.0	41.6	±3.7	0.057

Data presented as Mean ± S.E.M. for normally distributed values or median (Q1–Q3) for not-normally distributed values.

**Table 2:** Results of biological processes of differentially methylated genes-generated in Cytoscape software through BinGo plugin.

Description	Corr *p*-Value	X	*n*	Regulation	Genes
lipid metabolic process	9.47 × 10^−10^	35	834	Hypermethylated	*ABCA1; ACOXL; ACOX3; ATP8B1; CIDEA; CPT1A; CYP51A1; CYP26C1; CYP24A1; CYP46A1; CYP26B1; CYP27B1; DECR1; DHCR24; ELOVL6; FADS2; GATA6; LIPG; PPARA; PRKAR2B; SCD; VLDLR*
				Hypomethylated	*ACSL3; ACSS2; ADIPOR1; ATP8B1; CPT1A; CPT1B; CPT1C; CYP26A1; ELOVL4; ELOVL6; ELOVL7; GATA6; LPIN1; PDE3A; PRKAA1; PRKAB2; SLC27A1;*
lipid localization	9.18 × 10^−11^	17	151	Hypermethylated	*ABCA1; ABCG4; ATP8A2; ATP8B1; ATP10A; ATP10D; ATP11A; CIDEA; LIPG; PPARA; VLDLR;*
				Hypomethylated	*ATP8B1; ATP8B3; ATP9A; ATP9B; ATP11A; CPT1B; SLC27A1; STARD4;*
lipid transport	2.46 × 10^−10^	16	139	Hypermethylated	*ABCA1; ABCG4; ATP8A2; ATP8B1; ATP10D; ATP10A; ATP11A; LIPG; PPARA; VLDLR;*
				Hypomethylated	*ATP8B1; ATP8B3; ATP9A; ATP9B; ATP10A; CPT1B; SLC27A1; STARD4;*
regulation of lipid storage	1.48 × 10^−2^	3	27	Hypermethylated	*ABCA1; CIDEA; PPARA*
				Hypomethylated	
fatty acid metabolic process	1.79 × 10^−10^	19	209	Hypermethylated	*ACOX3; CPT1A; DECR1; ELOVL6; FADS2; ACOXL; SCD; PPARA*
				Hypomethylated	*ACSL3; ADIPOR1; CPT1A; CPT1B; CPT1C; ELOVL4; ELOVL6; ELOVL7; LPIN1; PRKAA1; PRKAB2; PRKAR2B; SLC27A1*
regulation of fatty acid oxidation	2.33 × 10^−5^	6	33	Hypermethylated	*CPT1A; PPARA*
				Hypomethylated	*CPT1B; IRS1; PRKAA1; PRKAB2;*
regulation of sequestering of triglyceride	1.74 × 10^−2^	2	9	Hypermethylated	*CIDEA; PPARA*
				Hypomethylated	
cholesterol metabolic process	1.66 × 10^−2^	5	91	Hypermethylated	*ABCA1; CYP46A1; CYP51A1; DHCR24; VLDLR*
				Hypomethylated	
regulation of insulin secretion	3.26 × 10^−5^	7	54	Hypermethylated	*ADRA2A; GIPR; NEUROD1; PFKM; TCF7L2;*
				Hypomethylated	*ADRA2A; CPT1A; IRS1;*
response to insulin stimulus	2.86 × 10^−6^	11	123	Hypermethylated	*IGFBP1; IGF1R; PDK1; PRKCI; PPARA; SLC2A8; VLDLR*
				Hypomethylated	*INSR; IRS1; SLC27A1; SOCS3;*
					
insulin receptor signaling pathway	4.44 × 10^−4^	5	36	Hypermethylated	*IGFBP1; IGF1R; SLC2A8*
				Hypomethylated	*INSR; IRS1*
glucose homeostasis	8.11 × 10^−9^	10	51	Hypermethylated	*BAD; FOXO3; NEUROD1; PDX1; PFKM; SLC2A4; TCF7L2;*
				Hypomethylated	*ADRA2A; INSR; IRS1*
response to glucose stimulus	6.03 × 10^−4^	6	62	Hypermethylated	*ADRA2A; GIPR; NEUROD1; TCF7L2*
				Hypomethylated	*ADRA2A; INSR; IRS1;*
glucose metabolic process	7.96 × 10^−5^	10	146	Hypermethylated	*ATF4; BAD; CPT1A; PDK1; PDX1; PFKM; SLC2A8*
				Hypomethylated	*PDK2; PFKL; PFKP*
glucose transport	1.60 × 10^−3^	4	27	Hypermethylated	*SLC2A4; SLC2A5; SLC2A8; KLF15;*
				Hypomethylated	*SLC2A5*
regulation of glucose transport	3.42 × 10^−3^	4	34	Hypermethylated	*PRKCI; PRKCZ*
				Hypomethylated	*INSR; IRS1; PRKCZ*
glycolysis	3.72 × 10^−2^	3	42	Hypermethylated	*PFKM*
				Hypomethylated	*PFKL; PFKP*
response to nutrient levels	8.47 × 10^−14^	24	243	Hypermethylated	*BMP7; BMPR2; CYP24A1; CYP27B1; GIPR; IGF1R; LIPG; PPARA; RARA; WNT3; WNT3A; VLDLR*
				Hypomethylated	*ACSL3; BMP2; BMP4; BMPR2; CEBPA; INSR; PDGFA; RARA; RPTOR; SOCS3; SOD1; WNT3A; WNT7B; WNT9A; WNT9B*
fibroblast growth factor receptor signalling pathway	1.35 × 10^−6^	7	33	Hypermethylated	*FGF3; FGF5; FGF9; FGFR1; FRS2*
				Hypomethylated	*FGF3; FGFR1; FGFR3; KLB*
positive regulation of MAPKKK cascade by fibroblast growth factor receptor signalling pathway	6.41 × 10^−3^	2	5	Hypermethylated	*FGFR1*
				Hypomethylated	*KLB; FGFR1*
fat cell differentiation	2.86 × 10^−7^	9	56	Hypermethylated	*ADRB1; CTBP2; PRDM16; SLC2A4; TCF7L2;*
				Hypomethylated	*CEBPA; CEBPB; CTBP1; CTBP2; PRDM16*
white fat cell differentiation	1.44 × 10^−2^	2	8	Hypermethylated	*CTBP2*
				Hypomethylated	*CTBP1; CTBP2*
brown fat cell differentiation	8.44 × 10^−5^	5	25	Hypermethylated	*ADRB1; PRDM16; SLC2A4*
				Hypomethylated	*CEBPA; CEBPB; PRDM16*
positive regulation of vascular endothelial growth factor receptor signalling pathway	1.95 × 10^−3^	3	12	Hypermethylated	*FGF9;*
				Hypomethylated	*FLT1; VEGFA*
response to oxygen levels	2.87 × 10^−5^	11	158	Hypermethylated	*ATP1B1; PDGFRA; PPARA; SLC2A8; VLDLR*
				Hypomethylated	*ANGPTL4; ATP1B; FLT1; PDGFA; PDGFRA; PRKAA1; SOCS3; VEGFA*
response to hypoxia	9.26 × 10^−5^	10	149	Hypermethylated	*ATP1B1; PPARA; SLC2A8; VEGFA*
				Hypomethylated	*ANGPTL4; ATP1B1; FLT1; PDGFA; PRKAA1; SOCS3; VLDLR*
vascular process in circulatory system	1.24 × 10^−2^	4	51	Hypermethylated	*ADRB1*
				Hypomethylated	*FGFBP3; SOD1; VEGFA*
positive regulation of canonical Wnt receptor signalling pathway	8.48 × 10^−4^	3	9	Hypermethylated	*WNT3A; WNT7A;*
				Hypomethylated	*WNT2B;*
regulation of ossification	9.96 × 10^−7^	10	86	Hypermethylated	*BMP6; BMP7; BMPR2; CYP27B1; ESRRA; GNAS; TFAP2A*
				Hypomethylated	*BMP2; BMP4; BMPR2; GNAS; WNT7B; TFAP2A*
regulation of bone mineralization	1.35 × 10^−6^	7	33	Hypermethylated	*BMPR2; BMP7; BMP6; CYP27B1; TFAP2A;*
				Hypomethylated	*BMP2; BMP4; BMPR2; TFAP2A;*
positive regulation of osteoblast differentiation	2.68 × 10^−7^	7	26	Hypermethylated	*BMPR2; GNAS*
				Hypomethylated	*BMP2; BMP4; BMPR2; GNAS; WNT7B*
generation of precursor metabolites and energy	5.08 × 10^−15^	28	312	Hypermethylated	*ATP6V1B2; ATP6V1C1; ATP6V1E1; FADS2; GIPR; GNAS; NDUFAF1; NDUFA6; NDUFA8; NDUFA9; NDUFA13; NDUFB1; NDUFB2; NDUFB5; NDUFB7; NDUFS1; NDUFS2; NDUFV2; PDX1; PFKM*
				Hypomethylated	*ATP5D; ATP5H; CEBPA; COX7A1; GNAS; NDUFA11; NDUFV3; PFKL; PFKP*
mitochondrial electron transport, NADH to ubiquinone	3.52 × 10^−12^	12	43	Hypermethylated	*NDUFA6; NDUFA8; NDUFA9; NDUFB1; NDUFB2; NDUFB5; NDUFB7; NDUFAF1; NDUFS1; NDUFS2; NDUFV2*
				Hypomethylated	*NDUFV3;*
ATP synthesis coupled electron transport	9.12 × 10^−11^	12	57	Hypermethylated	*NDUFA6; NDUFA8; NDUFA9; NDUFB1; NDUFB2; NDUFB5; NDUFB7; NDUFAF1; NDUFS1; NDUFS2; NDUFV2*
				Hypomethylated	*NDUFV3;*
mitochondrial ATP synthesis coupled electron transport	9.12 × 10^−11^	12	57	Hypermethylated	*NDUFA6; NDUFA8; NDUFA9; NDUFB1; NDUFB2; NDUFB5; NDUFB7; NDUFAF1; NDUFS1; NDUFS2; NDUFV2*
				Hypomethylated	*NDUFV3;*
	
response to retinoic acid	2.86 × 10^−7^	9	56	Hypermethylated	*WNT3; WNT3A; RARA;*
				Hypomethylated	*BMP2; BMP4; PDGFA; RARA; WNT3A; WNT7B; WNT9A; WNT9B;*
regulation of striated muscle tissue development	1.16 × 10^−2^	4	50	Hypermethylated	*FGF9; TBX5; FGFR1*
				Hypomethylated	*BMP4; FGFR1*
regulation of apoptosis	6.59 × 10^−5^	26	852	Hypermethylated	*BAD; BMP7; CEBPB; CIDEA; CFLAR; DHCR24; FGFR1; FOXO3; GATA6; IGF1R; IL7; MYO18A; NDUFA13; NEUROD1; PRKCI; PRKCZ; TBX5; TCF7L2*
				Hypomethylated	*ANGPTL4; BMP2; BMP4; FGFR1; GATA6; MYO18A; PDE3A; PRKCE; PRKCZ; SOCS3; SOD1; VEGFA*

**Table 3:** Results on identified differentially methylated transcription factors genes known to regulate FGF21 expression.

TF (Transcription Factor)	DNA Methylation Status	Region	Selected Regulated Genes ^a^	Reference
**PPARA**	Hypermethylated (2 probes)	Promoter	ACADVL, ACOT7, ACOT8, CYP26A1, FABP1, FBP2, FGF9, FGF21, GAPDH, HNF1A, HSD17B8, IGF2BP1, IL10, IL4, LPL, LRP1, MAP2K3, PDGFB, PPARGC1A, PRDM16, VEGFA	[33]
**RORA**	Hypermethylated (3 probes)	Promoter: CTCF binding sites	ABCA1, ACOXL, CYP26A1, FGF9, FGF21, IL7, KLF4	[11,34]
**ATF4**	Hypermethylated (1 probe)	Promoter	ABCA2, CPT1B, CYP26A1, ELOVL5, FGF21, GNAS, NDUFA10, S1PR2, SREBF2	[35,36]
**KLF15**	Hypermethylated (1 probe)	Promoter	FGF21, NR3C1, PPARA	[13]
**NR3C1**	Hypermethylated (1 probe) Hypomethylated (1 probe)	Promoter	BMPR2, CD36, CYP26A1, ELOVL6, ESRRA, FGF9, FGF21, IL10, PDGFRA, RORA, SCD, SLC2A2,	[37]

^a^ - Selection of targets genes based on TRANSFAC Curated Transcription Factor Targets database [38] and relevant publications.

**Table 4:** Results of the expression of miRNAs by quantitative PCR-TLDA (TaqMan low density array) arrays in leukocytes in the high FGF21 versus low FGF21 group.

miRNA Name	adj.*p*	FC	Selected Targets
**hsa-miR-133a-3p**	0.0477	−3.1	FGFR1
			VEGFA
			PRDM16
**hsa-miR-185-5p**	0.0496	−2.6	FGFR1
			VEGFA
**hsa-miR-200c-3p**	0.0347	−2.2	FGFR1
			VEGFA
			FLT1
			KDR
			TCF7L2
**hsa-miR-875-5p**	0.0052	13	ADIPOQ
			CPT1A
			IRS1
			LEP
			LIPA
			SLC2A2
			SLC2A3
			SLC2A4
			BMP8A
			BMPR1A

## Data Availability

Data from the BIOCLAIMS study are deposited in Nutritional Phenotype Database http://www.dbnp.org/nutritional-instance/ (accessed on 19 January 2021)). The data of differentially methylated CpGs probes presented in this study are available in Supplementary materials.

## Supplementary Materials

The following are available online at https://www.mdpi.com/2073-4425/12/2/307/s1, Figure S1: Graph of a biological network of identified as differentially methylated genes created by the ClueGO plugin in Cytoscape software, Table S1: Characteristics of subjects selected for epigenetics study, Table S2: Detail results of identified differentially methylated CpGs probes.
