# Supplementary material for: Epigenetic Regulation of Processes Related to High Level of Fibroblast Growth Factor 21 in Obese Subjects
Source: Genes (Basel). 2021 Feb 21;12(2):307. doi: 10.3390/genes12020307 (PMC7926457; doi:10.3390/genes12020307)
Supplement: Supplementary file 1 [file genes-12-00307-s001.zip › genes-1098573-supplementary/Figure S1.docx]

Figure S1. Graph of a biological network of identified as differentially methylated genes created by the ClueGO plugin in Cytoscape software.

Genes which are hypermethylated are marked as
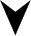
 and colored red, hypomethylated as circles and colored black.
