# Supplementary material for: Epigenetic Regulation of Processes Related to High Level of Fibroblast Growth Factor 21 in Obese Subjects
Source: Genes (Basel). 2021 Feb 21;12(2):307. doi: 10.3390/genes12020307 (PMC7926457; doi:10.3390/genes12020307)
Supplement: Supplementary file 1 [file genes-12-00307-s001.zip › genes-1098573-supplementary/Supplementary_Table S1.docx]

Table S1. Characteristics of subjects selected for epigenetics study

|  | High FGF21 | | Low FGF21 | | P |
| --- | --- | --- | --- | --- | --- |
| Sex. F/M (%) | 63 | | 63 | | Ns |
| Age (years) | 51.2 | ± 11.3 | 38.1 | ± 9.3 | 0.025 |
| BMI (kg/m2) | 36.5 | ± 5.9 | 33.1 | ± 1.5 | 0.21 |
| Adipose tissue mass (%) | 41.75 | (32.22-45.58) | 36.05 | (33.82-40.32) | 0.356 |
| WHR | 0.92 | (0.84-0.99) | 0.83 | (0.79-0.89) | 0.143 |
| Waist circumference (cm) | 114 | ± 11.2 | 97 | ± 8.2 | 0.010 |
| Systolic blood pressure (mmHg) | 135.7 | ± 9.42 | 122.4 | ± 11.22 | 0.030 |
| Diastolic blood pressure (mmHg) | 86.0 | ± 4.9 | 79.7 | ± 3.89 | 0.013 |
| Fasting glucose (mmol/L) | 5.65 | (5.04-5.85) | 4.95 | (4.90-5.37) | 0.303 |
| Insulin (µIU/mL) | 17.4 | ± 5.9 | 11.9 | ± 5.7 | 0.09 |
| HOMA-IR | 3.84 | (3.21-4.0) | 2.51 | (1.94-2.98) | 0.175 |
| VEGF (pg/mL) | 430.7 | ± 244.0 | 357.8 | ± 144.3 | 0.460 |
| Total Cholesterol (mmol/L) | 6.5 | ± 1.9 | 5.1 | ± 0.8 | 0.062 |
| HDL cholesterol (mmol/L) | 1.5 | ± 0.5 | 1.3 | ± 0.2 | 0.159 |
| LDL cholesterol (mmol/L) | 4.2 | ± 1.4 | 3.3 | ± 0.7 | 0.108 |
| TG (mmol/L) | 1.8 | ± 1.2 | 1.2 | ± 0.8 | 0.321 |
| GIP (pg/mL) | 30.11 | (20.72-44.32) | 23.87 | (15.34-33.20) | 0.481 |
| ALT (U/L) | 24.3 | ± 15.8 | 13.0 | ± 4.2 | 0.046 |
| GGT (U/L) | 64.8 | ± 102.7 | 15.4 | ± 6.6 | 0.143 |
| Leptin (ng/mL) | 25.71 | (15.51-52.72) | 27.15 | (18.96-32.89) | 0.957 |
| Adiponectin (µg/mL) | 4.2 | (3.8-7.7) | 7.6 | (7.4-8.3) | 0.255 |
| Resitin (ng/mL) | 10.3 | ± 5.8 | 10.3 | ± 2.3 | 0.598 |
| Visfatin (ng/mL) | 1.2 | ± 0.9 | 0.8 | ± 0.3 | 0.205 |
| **FGF21 (pg/mL)** | **246.8** | **(237.9-272.0)** | **129.7** | **(51.1 -152.95)** | **0.0014** |
| Irisin (µg/mL) | 5.5 | ± 2.5 | 5.4 | ± 1.4 | 0.916 |
| Myostatin (ng/mL) | 20.5 | (18.72-24.60) | 23.3 | (17.72-27.32) | 0.787 |
| IL-6 (pg/mL) | 1.63 | (0.55-1.20) | 0.93 | (0.98-2.21) | 0.116 |
| hs-CRP (mg/mL) | 3.78 | (1.75-5.22) | 1.07 | (0.26-2.33) | 0.035 |
| MCP1 (pg/mL) | 411.0 | ± 211.3 | 325.8 | ± 118.8 | 0.315 |
| sVCAM-1 (ng/mL) | 676.3 | ± 198.7 | 666.8 | ± 158.1 | 0.917 |
| sPECAM-1 (ng/mL) | 77.5 | ± 15.5 | 65.7 | ± 14.1 | 0.142 |
| sEselectin (pg/mL) | 63.2 | ± 39.9 | 35.7 | ± 17.8 | 0.076 |
| Fasting NEFAs (mmol/L) | 0.688 | ± 0.179 | 0.701 | ± 0.238 | 0.912 |
| Total fatty acids (µg/mL) | 4038.8 | ± 1015.8 | 3488.2 | ± 1270.4 | 0.392 |
| Saturated fatty acids (%) | 33.3 | ± 0.9 | 32.6 | ± 1.8 | 0.46 |
| Monounsaturated fatty acids (%) | 28.6 | ± 5.1 | 26.9 | ± 5.1 | 0.54 |
| Polyunsaturated fatty acids (%) | 38.1 | ± 5.1 | 40.4 | ± 6.5 | 0.48 |
