# Supplementary material for: Epigenetic Regulation of Processes Related to High Level of Fibroblast Growth Factor 21 in Obese Subjects
Source: Genes (Basel). 2021 Feb 21;12(2):307. doi: 10.3390/genes12020307 (PMC7926457; doi:10.3390/genes12020307)
Supplement: Supplementary file 1 [file genes-12-00307-s001.zip › genes-1098573-supplementary/Supplementary_Table S2.docx]

Table S2.Detail results of identified differentially methylated CpGs probes.

|  | Gene symbol | Gene Name | Unique ID | Genomic location of the methylated DNA fragment (according to hg38, GRCh38.p12) | q-value | p-value | FC (abs) | Regulation | Map Location |
| --- | --- | --- | --- | --- | --- | --- | --- | --- | --- |
| 1 | ABCA1 | ATP-binding cassette, sub-family A (ABC1), member 1 | A_17_P06870160 | chr9:104927909-104927959 | 1.30E-03 | 1.52E-05 | 3.77 | Hypermethylated | Promoter |
| 2 | ABCG4 | ATP-binding cassette, sub-family G (WHITE), member 4 | A_17_P28493790 | chr11:119149878-119149922 | 4.34E-03 | 1.34E-04 | 1.80 | Hypermethylated | Promoter |
| 3 | ACOX3 | acyl-CoA oxidase 3, pristanoyl | A_17_P23068206 | chr4:8373342-8373386 | 3.42E-03 | 8.83E-05 | 2.28 | Hypomethylated | Inside/intronic |
| 4 | ACOX3 | acyl-CoA oxidase 3, pristanoyl | A_17_P23068207 | chr4:8373418-8373462 | 9.73E-04 | 8.69E-06 | 2.94 | Hypomethylated | Inside/intronic |
| 5 | ACOX3 | acyl-CoA oxidase 3, pristanoyl | A_17_P15542410 | chr4:8375142-8375186 | 3.41E-03 | 8.82E-05 | 1.80 | Hypomethylated | Inside/intronic |
| 6 | ACOX3 | acyl-CoA oxidase 3, pristanoyl | A_17_P02918075 | chr4:8386646-8386695 | 2.56E-03 | 5.21E-05 | 1.89 | Hypomethylated | Inside/intronic |
| 7 | ACOX3 | acyl-CoA oxidase 3, pristanoyl | A_17_P15542505 | chr4:8428208-8428252 | 5.40E-03 | 1.93E-04 | 1.51 | Hypermethylated | Promoter |
| 8 | ACOXL | acyl-CoA oxidase-like | A_17_P01402624 | chr2:111117521-111117565 | 9.97E-03 | 5.47E-04 | 2.10 | Hypermethylated | Promoter/CTCF binding site |
| 9 | ACSL3 | acyl-CoA synthetase long-chain family member 3 | A_17_P22045295 | chr2:222861541-222861585 | 1.14E-03 | 1.18E-05 | 3.00 | Hypomethylated | Promoter |
| 10 | ACSS2 | acyl-CoA synthetase short-chain family member 2 | A_17_P11142898 | chr20:34877016-34877060 | 7.12E-03 | 3.10E-04 | 1.45 | Hypomethylated | Promoter |
| 11 | ACSS3 | acyl-CoA synthetase short-chain family member 3 | A_17_P08484053 | chr12:81077766-81077814 | 1.89E-03 | 3.03E-05 | 3.69 | Hypermethylated | Promoter |
| 12 | ACSS3 | acyl-CoA synthetase short-chain family member 3 | A_17_P08484056 | chr12:81078041-81078085 | 5.33E-03 | 1.88E-04 | 1.55 | Hypermethylated | Promoter |
| 13 | ADIPOR1 | adiponectin receptor 1 | A_17_P00735113 | chr1:202957832-202957876 | 3.40E-03 | 8.73E-05 | 1.95 | Hypomethylated | Promoter |
| 14 | ADRA1D | adrenergic, alpha-1D-, receptor | A_17_P11028377 | chr20:4221522-4221568 | 6.44E-03 | 2.58E-04 | 3.72 | Hypermethylated | Promoter/CTCF binding site |
| 15 | ADRA2A | adrenergic, alpha-2A-, receptor | A_17_P27870064 | chr10:111077099-111077143 | 2.42E-03 | 4.74E-05 | 1.80 | Hypermethylated | Promoter |
| 16 | ADRA2A | adrenergic, alpha-2A-, receptor | A_17_P27870085 | chr10:111079329-111079373 | 8.02E-03 | 3.79E-04 | 1.90 | Hypomethylated | Promoter |
| 17 | ADRB1 | adrenergic, beta-1-, receptor | A_17_P16386028 | chr10:114045176-114045220 | 6.51E-03 | 2.64E-04 | 1.81 | Hypermethylated | Promoter |
| 18 | ANGPTL4 | angiopoietin-like 4 | A_17_P10875677 | chr19:8367366-8367410 | 2.18E-03 | 3.93E-05 | 2.25 | Hypomethylated | Promoter |
| 19 | ANGPTL4 | angiopoietin-like 4 | A_17_P10875678 | chr19:8367419-8367463 | 9.79E-03 | 5.31E-04 | 2.47 | Hypomethylated | Promoter |
| 20 | ATF4 | activating transcription factor 4 (tax-responsive enhancer element B67) | A_17_P17284432 | chr22:39520563-39520607 | 1.32E-04 | 8.70E-08 | 3.77 | Hypermethylated | Promoter |
| 21 | ATP10A | ATPase, class V, type 10A | A_17_P09546555 | chr15:25713720-25713764 | 9.70E-03 | 5.22E-04 | 1.65 | Hypomethylated | Inside/exonic |
| 22 | ATP10A | ATPase, class V, type 10A | A_17_P09547302 | chr15:25862306-25862350 | 3.99E-03 | 1.16E-04 | 1.52 | Hypermethylated | Promoter |
| 23 | ATP10D | ATPase, class V, type 10D | A_17_P03087687 | chr4:47485431-47485482 | 1.83E-04 | 2.18E-07 | 6.71 | Hypermethylated | Promoter |
| 24 | ATP11A | ATPase, class VI, type 11A | A_17_P16673308 | chr13:112728346-112728390 | 2.89E-04 | 7.13E-07 | 2.86 | Hypomethylated | Inside/intronic |
| 25 | ATP11A | ATPase, class VI, type 11A | A_17_P16673309 | chr13:112728451-112728495 | 7.81E-03 | 3.64E-04 | 3.46 | Hypomethylated | Inside/intronic |
| 26 | ATP11A | ATPase, class VI, type 11A | A_17_P16673310 | chr13:112728549-112728593 | 2.87E-03 | 6.41E-05 | 4.01 | Hypomethylated | Inside/intronic |
| 27 | ATP11A | ATPase, class VI, type 11A | A_17_P16673311 | chr13:112728628-112728672 | 2.47E-03 | 4.90E-05 | 3.64 | Hypomethylated | Inside/intronic |
| 28 | ATP11A | ATPase, class VI, type 11A | A_17_P16673497 | chr13:112809927-112809971 | 8.45E-03 | 4.15E-04 | 2.53 | Hypomethylated | Inside/intronic |
| 29 | ATP11A | ATPase, class VI, type 11A | A_17_P29595447 | chr13:112819660-112819719 | 9.34E-03 | 4.89E-04 | 4.26 | Hypermethylated | Inside/intronic |
| 30 | ATP11A | ATPase, class VI, type 11A | A_17_P09145560 | chr13:112832501-112832545 | 8.73E-03 | 4.35E-04 | 1.94 | Hypomethylated | Inside/intronic |
| 31 | ATP11A | ATPase, class VI, type 11A | A_17_P16673527 | chr13:112833024-112833068 | 7.69E-03 | 3.54E-04 | 1.90 | Hypomethylated | Inside/intronic |
| 32 | ATP13A2 | ATPase type 13A2 | A_17_P15032774 | chr1:17011267-17011311 | 7.46E-04 | 5.15E-06 | 2.19 | Hypermethylated | Promoter/ CTCF binding site |
| 33 | ATP1B1 | ATPase, Na+/K+ transporting, beta 1 polypeptide | A_17_P00584594 | chr1:169105579-169105626 | 5.09E-03 | 1.75E-04 | 2.62 | Hypermethylated | Promoter |
| 34 | ATP1B1 | ATPase, Na+/K+ transporting, beta 1 polypeptide | A_17_P15142167 | chr1:169105747-169105791 | 8.91E-03 | 4.52E-04 | 2.42 | Hypomethylated | Promoter |
| 35 | ATP1B2 | ATPase, Na+/K+ transporting, beta 2 polypeptide | A_17_P10214357 | chr17:7651519-7651563 | 7.59E-03 | 3.46E-04 | 2.24 | Hypermethylated | Promoter |
| 36 | ATP1B3 | ATPase, Na+/K+ transporting, beta 3 polypeptide | A_17_P02631358 | chr3:141877373-141877417 | 8.97E-03 | 4.57E-04 | 2.08 | Hypermethylated | Promoter |
| 37 | ATP2A3 | ATPase, Ca++ transporting, ubiquitous | A_17_P16937165 | chr17:3944669-3944713 | 4.54E-03 | 1.45E-04 | 1.90 | Hypomethylated | Promoter/ CTCF binding site |
| 38 | ATP2B2 | ATPase, Ca++ transporting, plasma membrane 2 | A_17_P15411829 | chr3:10358884-10358928 | 3.20E-03 | 7.82E-05 | 1.54 | Hypomethylated | Inside/exonic |
| 39 | ATP5D | ATP synthase, H+ transporting, mitochondrial F1 complex, delta subunit | A_17_P17094060 | chr19:1240297-1240341 | 1.15E-03 | 1.19E-05 | 2.13 | Hypomethylated | Promoter/CTCF binding site |
| 40 | ATP5H | ATP synthase, H+ transporting, mitochondrial Fo complex, subunit d | A_17_P10464878 | chr17:75046500-75046544 | 5.61E-03 | 2.05E-04 | 1.83 | Hypomethylated | Promoter |
| 41 | ATP5J2-PTCD1 | ATP5J2-PTCD1 readthrough | A_17_P25896043 | chr7:99466014-99466058 | 7.12E-04 | 4.76E-06 | 1.92 | Hypermethylated | Promoter |
| 42 | ATP6V1B2 | ATPase, H+ transporting, lysosomal 56/58kDa, V1 subunit B2 | A_17_P05995027 | chr8:20197542-20197590 | 7.50E-03 | 3.39E-04 | 2.02 | Hypermethylated | Promoter |
| 43 | ATP6V1B2 | ATPase, H+ transporting, lysosomal 56/58kDa, V1 subunit B2 | A_17_P05995029 | chr8:20197672-20197726 | 8.78E-03 | 4.41E-04 | 4.57 | Hypermethylated | Promoter |
| 44 | ATP6V1C1 | ATPase, H+ transporting, lysosomal 42kDa, V1 subunit C1 | A_17_P06346553 | chr8:103021064-103021108 | 3.67E-03 | 1.00E-04 | 1.65 | Hypermethylated | Promoter |
| 45 | ATP6V1E1 | ATPase, H+ transporting, lysosomal 31kDa, V1 subunit E1 | A_17_P17251703 | chr22:17628812-17628856 | 1.77E-03 | 2.71E-05 | 1.64 | Hypermethylated | Promoter |
| 46 | ATP8A2 | ATPase, aminophospholipid transporter, class I, type 8A, member 2 | A_17_P29175785 | chr13:25468977-25469024 | 1.39E-03 | 1.72E-05 | 2.09 | Hypermethylated | Promoter |
| 47 | ATP8B1 | ATPase, aminophospholipid transporter, class I, type 8B, member 1 | A_17_P17072889 | chr18:57648594-57648638 | 4.77E-03 | 1.57E-04 | 1.94 | Hypomethylated | Inside/exonic |
| 48 | ATP8B1 | ATPase, aminophospholipid transporter, class I, type 8B, member 1 | A_17_P10738103 | chr18:57803831-57803876 | 2.04E-03 | 3.50E-05 | 2.29 | Hypermethylated | Promoter |
| 49 | ATP8B3 | ATPase, aminophospholipid transporter, class I, type 8B, member 3 | A_17_P17095530 | chr19:1800011-1800055 | 1.80E-04 | 2.11E-07 | 2.27 | Hypomethylated | Inside/exonic |
| 50 | ATP9A | ATPase, class II, type 9A | A_17_P31860463 | chr20:51768319-51768363 | 5.86E-03 | 2.21E-04 | 1.72 | Hypomethylated | Promoter |
| 51 | ATP9B | ATPase, class II, type 9B | A_17_P17088028 | chr18:79069195-79069239 | 4.33E-03 | 1.34E-04 | 1.53 | Hypomethylated | Promoter/CTCF binding site |
| 52 | ATP9B | ATPase, class II, type 9B | A_17_P17088389 | chr18:79365832-79365876 | 3.31E-03 | 8.36E-05 | 3.36 | Hypomethylated | Promoter flanking region |
| 53 | ATP9B | ATPase, class II, type 9B | A_17_P31449667 | chr18:79365873-79365917 | 2.32E-03 | 4.38E-05 | 3.09 | Hypomethylated | Promoter flanking region |
| 54 | ATPAF1 | ATP synthase mitochondrial F1 complex assembly factor 1 | A_17_P00179772 | chr1:46668724-46668783 | 7.11E-03 | 3.09E-04 | 2.57 | Hypomethylated | Promoter |
| 55 | ATPAF2 | ATP synthase mitochondrial F1 complex assembly factor 2 | A_17_P10257899 | chr17:18039031-18039075 | 9.39E-03 | 4.94E-04 | 2.33 | Hypomethylated | Promoter |
| 56 | BAD | BCL2-associated agonist of cell death | A_17_P07838284 | chr11:64270110-64270157 | 2.72E-03 | 5.84E-05 | 4.32 | Hypermethylated | Promoter |
| 57 | BAMBI | BMP and activin membrane-bound inhibitor homolog (Xenopus laevis) | A_17_P07149001 | chr10:28668670-28668720 | 8.35E-03 | 4.06E-04 | 2.25 | Hypermethylated | Promoter |
| 58 | BAMBI | BMP and activin membrane-bound inhibitor homolog (Xenopus laevis) | A_17_P27507998 | chr10:28668962-28669013 | 7.11E-04 | 4.74E-06 | 2.61 | Hypermethylated | Promoter |
| 59 | BMP2 | bone morphogenetic protein 2 | A_17_P17171056 | chr20:6768005-6768049 | 3.10E-03 | 7.35E-05 | 2.48 | Hypomethylated | Promoter |
| 60 | BMP4 | bone morphogenetic protein 4 | A_17_P16703720 | chr14:53946928-53946972 | 7.53E-03 | 3.41E-04 | 1.78 | Hypomethylated | Promoter/ CTCF binding site |
| 61 | BMP4 | bone morphogenetic protein 4 | A_17_P09299977 | chr14:53953855-53953899 | 8.73E-03 | 4.35E-04 | 2.85 | Hypomethylated | Promoter |
| 62 | BMP4 | bone morphogenetic protein 4 | A_17_P16703732 | chr14:53955230-53955274 | 4.95E-04 | 2.26E-06 | 2.13 | Hypomethylated | Promoter |
| 63 | BMP6 | bone morphogenetic protein 6 | A_17_P04536913 | chr6:7728828-7728883 | 6.98E-04 | 4.58E-06 | 2.17 | Hypermethylated | Promoter |
| 64 | BMP7 | bone morphogenetic protein 7 | A_17_P11233708 | chr20:57264338-57264390 | 7.01E-03 | 3.01E-04 | 2.12 | Hypermethylated | Promoter |
| 65 | BMP8A | bone morphogenetic protein 8a | A_17_P15051738 | chr1:39515853-39515897 | 2.79E-03 | 6.09E-05 | 2.26 | Hypomethylated | Promoter/ CTCF binding site |
| 66 | BMP8A | bone morphogenetic protein 8a | A_17_P15051381 | chr1:39516102-39516148 | 4.81E-03 | 1.60E-04 | 1.93 | Hypomethylated | Promoter/ CTCF binding site |
| 67 | BMPR2 | bone morphogenetic protein receptor, type II (serine/threonine kinase) | A_17_P01818830 | chr2:202375670-202375716 | 6.48E-03 | 2.61E-04 | 2.62 | Hypermethylated | Promoter/Trancription factor bindingsite |
| 68 | BMPR2 | bone morphogenetic protein receptor, type II (serine/threonine kinase) | A_17_P01818831 | chr2:202375746-202375790 | 6.65E-03 | 2.75E-04 | 1.67 | Hypomethylated | Promoter/Trancription factor binding site |
| 69 | BMPR2 | bone morphogenetic protein receptor, type II (serine/threonine kinase) | A_17_P15373719 | chr2:202376061-202376105 | 1.15E-03 | 1.20E-05 | 1.93 | Hypomethylated | Promoter/Trancription factor binding site |
| 70 | CEBPA | CCAAT/enhancer binding protein (C/EBP), alpha | A_17_P10940201 | chr19:33302208-33302252 | 5.70E-03 | 2.11E-04 | 1.98 | Hypomethylated | Promoter/CTCF binding site |
| 71 | CEBPB | CCAAT/enhancer binding protein (C/EBP), beta | A_17_P17198523 | chr20:50191985-50192029 | 6.55E-03 | 2.67E-04 | 2.77 | Hypomethylated | Promoter |
| 72 | CEBPD | CCAAT/enhancer binding protein (C/EBP), delta | A_17_P06101675 | chr8:47737848-47737892 | 5.13E-03 | 1.77E-04 | 2.23 | Hypomethylated | Promoter |
| 73 | CFLAR | CASP8 and FADD-like apoptosis regulator | A_17_P01813642 | chr2:201118623-201118676 | 4.96E-04 | 2.29E-06 | 2.57 | Hypermethylated | Promoter |
| 74 | CFLAR | CASP8 and FADD-like apoptosis regulator | A_17_P21938251 | chr2:201118997-201119041 | 1.35E-03 | 1.63E-05 | 2.48 | Hypermethylated | Promoter |
| 75 | CIDEA | cell death-inducing DFFA-like effector a | A_17_P17040435 | chr18:12254541-12254585 | 3.96E-03 | 1.15E-04 | 1.91 | Hypermethylated | Inside/exonic |
| 76 | COX7A1 | cytochrome c oxidase subunit VIIa polypeptide 1 (muscle) | A_17_P31577831 | chr19:36152282-36152326 | 7.04E-03 | 3.04E-04 | 1.62 | Hypomethylated | Promoter |
| 77 | CPT1A | carnitine palmitoyltransferase 1A (liver) | A_17_P28270730 | chr11:68841229-68841273 | 5.43E-03 | 1.94E-04 | 2.32 | Hypermethylated | Promoter |
| 78 | CPT1A | carnitine palmitoyltransferase 1A (liver) | A_17_P16467298 | chr11:68844216-68844260 | 1.56E-03 | 2.14E-05 | 1.80 | Hypermethylated | Promoter/ CTCF binding site |
| 79 | CPT1A | carnitine palmitoyltransferase 1A (liver) | A_17_P07855394 | chr11:68844279-68844326 | 5.27E-03 | 1.85E-04 | 1.71 | Hypomethylated | Promoter/ CTCF binding site |
| 80 | CPT1B | carnitine palmitoyltransferase 1B (muscle) | A_17_P11550999 | chr22:50577790-50577834 | 7.17E-03 | 3.14E-04 | 1.55 | Hypomethylated | Promoter |
| 81 | CPT1B | carnitine palmitoyltransferase 1B (muscle) | A_17_P17299250 | chr22:50578169-50578213 | 1.30E-03 | 1.53E-05 | 2.80 | Hypomethylated | Promoter |
| 82 | CPT1C | carnitine palmitoyltransferase 1C | A_17_P17152961 | chr19:49701492-49701537 | 4.07E-03 | 1.20E-04 | 2.47 | Hypermethylated | Inside/exonic |
| 83 | CPT1C | carnitine palmitoyltransferase 1C | A_17_P17152987 | chr19:49719651-49719695 | 1.59E-04 | 1.61E-07 | 2.26 | Hypomethylated | Promoter |
| 84 | CPT1C | carnitine palmitoyltransferase 1C | A_17_P31625307 | chr19:49719761-49719811 | 1.15E-03 | 1.20E-05 | 2.31 | Hypomethylated | Promoter |
| 85 | CPT1C | carnitine palmitoyltransferase 1C | A_17_P17152989 | chr19:49719862-49719906 | 1.51E-03 | 2.02E-05 | 2.02 | Hypomethylated | Promoter |
| 86 | CPT1C | carnitine palmitoyltransferase 1C | A_17_P17152990 | chr19:49719933-49719980 | 3.22E-03 | 7.91E-05 | 2.16 | Hypomethylated | Promoter |
| 87 | CPT1C | carnitine palmitoyltransferase 1C | A_17_P31625310 | chr19:49720023-49720067 | 3.38E-04 | 1.04E-06 | 2.17 | Hypomethylated | Promoter |
| 88 | CREB5 | cAMP responsive element binding protein 5 | A_17_P05384579 | chr7:28409327-28409386 | 6.91E-03 | 2.94E-04 | 2.64 | Hypermethylated | Promoter |
| 89 | CREB5 | cAMP responsive element binding protein 5 | A_17_P05384580 | chr7:28409398-28409447 | 3.34E-03 | 8.48E-05 | 1.84 | Hypermethylated | Promoter |
| 90 | CTBP1 | C-terminal binding protein 1 | A_17_P02888977 | chr4:1235361-1235420 | 6.08E-03 | 2.35E-04 | 2.37 | Hypomethylated | Inside/intronic |
| 91 | CTBP1 | C-terminal binding protein 1 | A_17_P15532548 | chr4:1240200-1240244 | 1.43E-03 | 1.80E-05 | 2.26 | Hypomethylated | Inside/intronic |
| 92 | CTBP2 | C-terminal binding protein 2 | A_17_P16393755 | chr10:124998001-124998045 | 4.35E-03 | 1.35E-04 | 2.15 | Hypomethylated | Inside/exonic |
| 93 | CTBP2 | C-terminal binding protein 2 | A_17_P07549591 | chr10:125151359-125151403 | 2.32E-03 | 4.37E-05 | 2.73 | Hypomethylated | Promoter |
| 94 | CTBP2 | C-terminal binding protein 2 | A_17_P27940477 | chr10:125160521-125160574 | 9.35E-03 | 4.90E-04 | 1.92 | Hypermethylated | Promoter |
| 95 | CYP20A1 | cytochrome P450, family 20, subfamily A, polypeptide 1 | A_17_P01821689 | chr2:203239456-203239507 | 6.05E-03 | 2.33E-04 | 2.29 | Hypermethylated | Promoter |
| 96 | CYP24A1 | cytochrome P450, family 24, subfamily A, polypeptide 1 | A_17_P31870791 | chr20:54173317-54173363 | 1.32E-03 | 1.57E-05 | 3.38 | Hypermethylated | Promoter |
| 97 | CYP26A1 | cytochrome P450, family 26, subfamily A, polypeptide 1 | A_17_P07397834 | chr10:93069181-93069225 | 5.54E-03 | 2.00E-04 | 2.21 | Hypomethylated | 3' UTR |
| 98 | CYP26A1 | cytochrome P450, family 26, subfamily A, polypeptide 1 | A_17_P07397863 | chr10:93074574-93074618 | 8.89E-03 | 4.49E-04 | 1.75 | Hypomethylated | Promoter |
| 99 | CYP26A1 | cytochrome P450, family 26, subfamily A, polypeptide 1 | A_17_P27787456 | chr10:93075052-93075097 | 7.01E-03 | 3.01E-04 | 1.60 | Hypermethylated | Inside/exonic |
| 100 | CYP26A1 | cytochrome P450, family 26, subfamily A, polypeptide 1 | A_17_P07397870 | chr10:93075424-93075468 | 1.10E-03 | 1.08E-05 | 1.85 | Hypomethylated | Promoter |
| 101 | CYP26B1 | cytochrome P450, family 26, subfamily B, polypeptide 1 | A_17_P01272077 | chr2:72144356-72144415 | 7.02E-03 | 3.03E-04 | 1.81 | Hypermethylated | Promoter/CTCF binding site |
| 102 | CYP26B1 | cytochrome P450, family 26, subfamily B, polypeptide 1 | A_17_P01272079 | chr2:72145310-72145355 | 3.22E-03 | 7.92E-05 | 3.18 | Hypermethylated | Promoter |
| 103 | CYP26C1 | cytochrome P450, family 26, subfamily C, polypeptide 1 | A_17_P07397792 | chr10:93061257-93061301 | 3.30E-03 | 8.32E-05 | 1.56 | Hypermethylated | Inside/exonic |
| 104 | CYP27B1 | cytochrome P450, family 27, subfamily B, polypeptide 1 | A_17_P28814522 | chr12:57766759-57766808 | 4.11E-03 | 1.23E-04 | 2.36 | Hypermethylated | Promoter/ CTCF binding site |
| 105 | CYP46A1 | cytochrome P450, family 46, subfamily A, polypeptide 1 | A_17_P16733457 | chr14:99730073-99730117 | 6.54E-03 | 2.66E-04 | 1.60 | Hypermethylated | CTCF binding site |
| 106 | CYP51A1 | cytochrome P450, family 51, subfamily A, polypeptide 1 | A_17_P15988656 | chr7:92133792-92133836 | 9.20E-03 | 4.77E-04 | 1.45 | Hypermethylated | Promoter |
| 107 | DECR1 | 2,4-dienoyl CoA reductase 1, mitochondrial | A_17_P06287198 | chr8:90001390-90001434 | 1.41E-03 | 1.76E-05 | 2.19 | Hypermethylated | Promoter |
| 108 | DHCR24 | 24-dehydrocholesterol reductase | A_17_P00210048 | chr1:54887488-54887532 | 9.67E-03 | 5.19E-04 | 1.88 | Hypermethylated | Promoter |
| 109 | DHRS7C | dehydrogenase/reductase (SDR family) member 7C | A_17_P10223508 | chr17:9771596-9771640 | 1.37E-03 | 1.67E-05 | 2.41 | Hypermethylated | Inside/exonic |
| 110 | ELOVL4 | elongation of very long chain fatty acids (FEN1/Elo2, SUR4/Elo3, yeast)-like 4 | A_17_P04845021 | chr6:79947466-79947510 | 9.41E-04 | 8.20E-06 | 3.23 | Hypomethylated | Promoter |
| 111 | ELOVL6 | ELOVL family member 6, elongation of long chain fatty acids (FEN1/Elo2, SUR4/Elo3-like, yeast) | A_17_P03345547 | chr4:110197496-110197554 | 6.66E-03 | 2.76E-04 | 2.33 | Hypermethylated | Promoter |
| 112 | ELOVL6 | ELOVL family member 6, elongation of long chain fatty acids (FEN1/Elo2, SUR4/Elo3-like, yeast) | A_17_P23504292 | chr4:110198791-110198835 | 9.35E-03 | 4.90E-04 | 1.66 | Hypomethylated | Promoter |
| 113 | ELOVL7 | ELOVL family member 7, elongation of long chain fatty acids (yeast) | A_17_P24136852 | chr5:60743754-60743798 | 1.98E-03 | 3.30E-05 | 1.91 | Hypomethylated | CTCF binding site |
| 114 | ESRRA | estrogen-related receptor alpha | A_17_P07838446 | chr11:64304996-64305040 | 1.01E-03 | 9.20E-06 | 1.90 | Hypermethylated | Promoter |
| 115 | ESRRA | estrogen-related receptor alpha | A_17_P28250727 | chr11:64306178-64306222 | 5.15E-03 | 1.78E-04 | 1.73 | Hypermethylated | Promoter |
| 116 | ESRRB | estrogen-related receptor beta | A_17_P16718081 | chr14:76376167-76376211 | 1.74E-03 | 2.62E-05 | 1.93 | Hypermethylated | Promoter |
| 117 | FADS2 | fatty acid desaturase 2 | A_17_P07829631 | chr11:61827529-61827573 | 3.57E-03 | 9.56E-05 | 1.57 | Hypermethylated | Promoter |
| 118 | FGF3 | fibroblast growth factor 3 | A_17_P07859406 | chr11:69817165-69817209 | 3.32E-04 | 1.01E-06 | 2.30 | Hypermethylated | Inside/intronic |
| 119 | FGF3 | fibroblast growth factor 3 | A_17_P16468591 | chr11:69817392-69817436 | 3.56E-03 | 9.48E-05 | 1.79 | Hypomethylated | Inside/intronic |
| 120 | FGF3 | fibroblast growth factor 3 | A_17_P16468597 | chr11:69818123-69818167 | 1.68E-03 | 2.45E-05 | 2.08 | Hypomethylated | Inside/intronic |
| 121 | FGF5 | fibroblast growth factor 5 | A_17_P23371242 | chr4:80266461-80266505 | 4.94E-03 | 1.67E-04 | 1.68 | Hypermethylated | Promoter |
| 122 | FGF9 | fibroblast growth factor 9 (glia-activating factor) | A_17_P16615919 | chr13:21671197-21671241 | 4.94E-03 | 1.67E-04 | 1.51 | Hypermethylated | Promoter |
| 123 | FGFBP3 | fibroblast growth factor binding protein 3 | A_17_P07393333 | chr10:91909024-91909068 | 9.51E-03 | 5.05E-04 | 1.72 | Hypomethylated | Promoter/ CTCF binding site |
| 124 | FGFR1 | fibroblast growth factor receptor 1 | A_17_P06079168 | chr8:38466107-38466160 | 1.09E-03 | 1.07E-05 | 2.72 | Hypermethylated | Promoter |
| 125 | FGFR1 | fibroblast growth factor receptor 1 | A_17_P06079182 | chr8:38468790-38468834 | 9.98E-03 | 5.47E-04 | 1.71 | Hypomethylated | Promoter/CTCF binding site |
| 126 | FGFR3 | fibroblast growth factor receptor 3 | A_17_P23037389 | chr4:1794436-1794480 | 1.34E-03 | 1.60E-05 | 1.71 | Hypomethylated | Promoter |
| 127 | FGFRL1 | fibroblast growth factor receptor-like 1 | A_17_P02888045 | chr4:1010592-1010636 | 2.21E-03 | 4.04E-05 | 3.36 | Hypermethylated | Promoter/CTCF binding site |
| 128 | FGFRL1 | fibroblast growth factor receptor-like 1 | A_17_P02888048 | chr4:1010946-1010990 | 4.11E-03 | 1.23E-04 | 2.26 | Hypomethylated | Promoter/CTCF binding site |
| 129 | FGFRL1 | fibroblast growth factor receptor-like 1 | A_17_P15532004 | chr4:1011126-1011170 | 7.12E-03 | 3.10E-04 | 1.85 | Hypomethylated | Promoter/CTCF binding site |
| 130 | FGFRL1 | fibroblast growth factor receptor-like 1 | A_17_P23033415 | chr4:1024592-1024636 | 9.45E-03 | 5.00E-04 | 2.11 | Hypomethylated | Inside/exonic |
| 131 | FLT1 | fms-related tyrosine kinase 1 (vascular endothelial growth factor/vascular permeability factor receptor) | A_17_P08743794 | chr13:28494632-28494676 | 3.21E-03 | 7.90E-05 | 2.68 | Hypomethylated | Promoter |
| 132 | FLT4 | fms-related tyrosine kinase 4 | A_17_P15773453 | chr5:180615351-180615395 | 5.61E-03 | 2.05E-04 | 2.87 | Hypomethylated | Enhancer |
| 133 | FLT4 | fms-related tyrosine kinase 4 | A_17_P15773455 | chr5:180615388-180615432 | 5.23E-03 | 1.83E-04 | 2.98 | Hypomethylated | Enhancer |
| 134 | FLT4 | fms-related tyrosine kinase 4 | A_17_P15773454 | chr5:180615466-180615510 | 2.40E-04 | 4.45E-07 | 2.73 | Hypomethylated | Inside/intronic |
| 135 | FNDC1 | fibronectin type III domain containing 1 | A_17_P15882192 | chr6:159168986-159169030 | 7.12E-03 | 3.10E-04 | 1.53 | Hypomethylated | Promoter/CTCF binding site |
| 136 | FNDC1 | fibronectin type III domain containing 1 | A_17_P25410667 | chr6:159169150-159169194 | 3.35E-03 | 8.53E-05 | 3.46 | Hypomethylated | Promoter/CTCF binding site |
| 137 | FNDC1 | fibronectin type III domain containing 1 | A_17_P15882262 | chr6:159233117-159233161 | 7.04E-03 | 3.04E-04 | 1.66 | Hypomethylated | Inside/exonic |
| 138 | FNDC3A | fibronectin type III domain containing 3A | A_17_P08838686 | chr13:48975283-48975336 | 1.28E-03 | 1.47E-05 | 2.47 | Hypermethylated | Promoter |
| 139 | FOXO3 | forkhead box O3 | A_17_P04972488 | chr6:108562006-108562050 | 3.08E-03 | 7.27E-05 | 2.09 | Hypermethylated | Promoter |
| 140 | FRS2 | fibroblast growth factor receptor substrate 2 | A_17_P08430291 | chr12:69470746-69470790 | 1.08E-03 | 1.05E-05 | 2.87 | Hypermethylated | Promoter |
| 141 | GATA6 | GATA binding protein 6 | A_17_P17048898 | chr18:22171103-22171147 | 6.26E-03 | 2.46E-04 | 2.13 | Hypomethylated | Promoter |
| 142 | GATA6 | GATA binding protein 6 | A_17_P31164252 | chr18:22171251-22171295 | 7.68E-03 | 3.53E-04 | 1.93 | Hypermethylated | Promoter |
| 143 | GATA6 | GATA binding protein 6 | A_17_P17048921 | chr18:22177068-22177112 | 3.42E-03 | 8.84E-05 | 2.06 | Hypomethylated | Promoter |
| 144 | GIPR | gastric inhibitory polypeptide receptor | A_17_P31610965 | chr19:45676973-45677017 | 4.22E-03 | 1.28E-04 | 1.68 | Hypermethylated | Promoter |
| 145 | GIPR | gastric inhibitory polypeptide receptor | A_17_P10975447 | chr19:45677507-45677551 | 1.80E-03 | 2.80E-05 | 4.02 | Hypermethylated | Promoter |
| 146 | GIPR | gastric inhibitory polypeptide receptor | A_17_P10975451 | chr19:45677889-45677945 | 1.32E-03 | 1.58E-05 | 2.73 | Hypermethylated | Promoter |
| 147 | GIPR | gastric inhibitory polypeptide receptor | A_17_P31610983 | chr19:45681890-45681934 | 4.68E-04 | 2.05E-06 | 1.91 | Hypermethylated | CTCF binding site |
| 148 | GNAS | GNAS complex locus | A_17_P11241691 | chr20:58839491-58839535 | 9.99E-03 | 5.49E-04 | 1.68 | Hypomethylated | Promoter/CTCF binding site |
| 149 | GNAS | GNAS complex locus | A_17_P11241797 | chr20:58852588-58852632 | 4.34E-03 | 1.34E-04 | 1.52 | Hypermethylated | Promoter/CTCF binding site |
| 150 | GNAS | GNAS complex locus | A_17_P11241804 | chr20:58853284-58853331 | 4.76E-03 | 1.56E-04 | 2.57 | Hypomethylated | CTCF binding site |
| 151 | GNAS | GNAS complex locus | A_17_P11242074 | chr20:58889647-58889699 | 8.81E-03 | 4.43E-04 | 1.44 | Hypomethylated | Promoter |
| 152 | GNAS | GNAS complex locus | A_17_P31892043 | chr20:58890085-58890132 | 3.60E-03 | 9.69E-05 | 1.85 | Hypomethylated | Promoter |
| 153 | GNAS | GNAS complex locus | A_17_P11242077 | chr20:58890887-58890934 | 9.10E-03 | 4.68E-04 | 2.26 | Hypermethylated | Promoter |
| 154 | GNAS | GNAS complex locus | A_17_P11242079 | chr20:58892244-58892288 | 2.41E-04 | 4.54E-07 | 2.30 | Hypomethylated | Promoter |
| 155 | IGF1R | insulin-like growth factor 1 receptor | A_17_P16831296 | chr15:98650763-98650807 | 9.99E-03 | 5.49E-04 | 1.63 | Hypermethylated | Promoter |
| 156 | IGF1R | insulin-like growth factor 1 receptor | A_17_P09865744 | chr15:98707618-98707662 | 8.43E-03 | 4.13E-04 | 1.46 | Hypomethylated | Inside/exonic |
| 157 | IGF1R | insulin-like growth factor 1 receptor | A_17_P09865745 | chr15:98707687-98707731 | 2.62E-03 | 5.48E-05 | 1.83 | Hypomethylated | Inside/exonic |
| 158 | IGF1R | insulin-like growth factor 1 receptor | A_17_P30369794 | chr15:98707825-98707878 | 4.97E-04 | 2.31E-06 | 3.84 | Hypomethylated | Inside/exonic |
| 159 | IGFBP1 | insulin-like growth factor binding protein 1 | A_17_P05462793 | chr7:45888344-45888394 | 3.13E-03 | 7.47E-05 | 2.08 | Hypermethylated | Promoter |
| 160 | IL7 | interleukin 7 | A_17_P06239845 | chr8:78805143-78805196 | 3.51E-03 | 9.25E-05 | 3.16 | Hypermethylated | Promoter |
| 161 | INSR | insulin receptor | A_17_P10871985 | chr19:7142812-7142856 | 2.91E-03 | 6.59E-05 | 2.03 | Hypomethylated | Inside/exonic |
| 162 | INSR | insulin receptor | A_17_P10872340 | chr19:7267441-7267495 | 4.39E-03 | 1.37E-04 | 2.10 | Hypomethylated | Inside/exonic |
| 163 | INSR | insulin receptor | A_17_P17103878 | chr19:7267515-7267562 | 6.25E-03 | 2.45E-04 | 1.87 | Hypomethylated | Inside/exonic |
| 164 | INSR | insulin receptor | A_17_P17103895 | chr19:7293446-7293490 | 1.89E-03 | 3.04E-05 | 1.91 | Hypomethylated | Promoter |
| 165 | INSR | insulin receptor | A_17_P31484530 | chr19:7294410-7294454 | 7.74E-03 | 3.58E-04 | 1.47 | Hypomethylated | Promoter/Trancription factor bindign site |
| 166 | INSRR | insulin receptor-related receptor | A_17_P15133559 | chr1:156845550-156845594 | 1.42E-03 | 1.78E-05 | 1.97 | Hypermethylated | Promoter Flanking region |
| 167 | IRS1 | insulin receptor substrate 1 | A_17_P15389136 | chr2:226791974-226792018 | 5.66E-03 | 2.07E-04 | 1.87 | Hypomethylated | Promoter |
| 168 | KLB | klotho beta | A_17_P23206429 | chr4:39446520-39446564 | 4.67E-03 | 1.51E-04 | 2.01 | Hypomethylated | Inside/exonic |
| 169 | KLF15 | Kruppel-like factor 15 | A_17_P02564844 | chr3:126356558-126356602 | 2.89E-03 | 6.50E-05 | 3.38 | Hypermethylated | Promoter |
| 170 | LIPG | lipase, endothelial | A_17_P10697216 | chr18:49561567-49561611 | 7.70E-03 | 3.55E-04 | 2.12 | Hypermethylated | Promoter |
| 171 | LIPG | lipase, endothelial | A_17_P17067308 | chr18:49561724-49561768 | 1.71E-03 | 2.54E-05 | 2.34 | Hypermethylated | Promoter |
| 172 | LPIN1 | lipin 1 | A_17_P00991171 | chr2:11746284-11746328 | 4.24E-04 | 1.67E-06 | 2.05 | Hypomethylated | Promoter |
| 173 | MRAP | melanocortin 2 receptor accessory protein | A_17_P11353998 | chr21:32300292-32300339 | 3.93E-03 | 1.14E-04 | 2.22 | Hypomethylated | CTCF binding site |
| 174 | MRAP | melanocortin 2 receptor accessory protein | A_17_P17231097 | chr21:32300393-32300437 | 4.34E-04 | 1.74E-06 | 6.27 | Hypomethylated | CTCF binding site |
| 175 | MRAP | melanocortin 2 receptor accessory protein | A_17_P11353999 | chr21:32300512-32300556 | 4.77E-05 | 3.92E-09 | 4.45 | Hypomethylated | Inside/intronic |
| 176 | MRAP | melanocortin 2 receptor accessory protein | A_17_P11354000 | chr21:32300587-32300631 | 1.25E-03 | 1.42E-05 | 3.12 | Hypomethylated | Inside/intronic |
| 177 | MRAP | melanocortin 2 receptor accessory protein | A_17_P11354001 | chr21:32300720-32300764 | 8.26E-03 | 3.99E-04 | 3.65 | Hypomethylated | Inside/intronic |
| 178 | MRAP | melanocortin 2 receptor accessory protein | A_17_P32013625 | chr21:32300812-32300856 | 1.54E-03 | 2.09E-05 | 2.82 | Hypomethylated | Inside/intronic |
| 179 | MYO18A | myosin XVIIIA | A_17_P10275754 | chr17:29166607-29166651 | 9.50E-04 | 8.35E-06 | 2.11 | Hypomethylated | Inside/exonic |
| 180 | MYO18A | myosin XVIIIA | A_17_P10275755 | chr17:29166680-29166724 | 6.63E-04 | 4.02E-06 | 1.93 | Hypomethylated | Inside/exonic |
| 181 | MYO18A | myosin XVIIIA | A_17_P10275756 | chr17:29166774-29166818 | 7.36E-03 | 3.28E-04 | 1.76 | Hypomethylated | Inside/exonic |
| 182 | MYO18A | myosin XVIIIA | A_17_P10275812 | chr17:29179812-29179856 | 7.07E-04 | 4.68E-06 | 2.28 | Hypermethylated | Promoter |
| 183 | NDUFA11 | NADH dehydrogenase (ubiquinone) 1 alpha subcomplex, 11, 14.7kDa | A_17_P31479703 | chr19:5903747-5903791 | 4.13E-03 | 1.24E-04 | 1.91 | Hypomethylated | Promoter |
| 184 | NDUFA13 | NADH dehydrogenase (ubiquinone) 1 alpha subcomplex, 13 | A_17_P10909550 | chr19:19516155-19516199 | 5.12E-03 | 1.76E-04 | 2.73 | Hypermethylated | Promoter |
| 185 | NDUFA4L2 | NADH dehydrogenase (ubiquinone) 1 alpha subcomplex, 4-like 2 | A_17_P16563112 | chr12:57238114-57238158 | 5.43E-03 | 1.94E-04 | 1.70 | Hypermethylated | Promoter/ CTCF binding site |
| 186 | NDUFA6 | NADH dehydrogenase (ubiquinone) 1 alpha subcomplex, 6, 14kDa | A_17_P11514380 | chr22:42090587-42090631 | 5.66E-03 | 2.08E-04 | 1.59 | Hypermethylated | Promoter |
| 187 | NDUFA8 | NADH dehydrogenase (ubiquinone) 1 alpha subcomplex, 8, 19kDa | A_17_P16260629 | chr9:122159577-122159621 | 1.08E-03 | 1.04E-05 | 2.49 | Hypermethylated | Promoter |
| 188 | NDUFA8 | NADH dehydrogenase (ubiquinone) 1 alpha subcomplex, 8, 19kDa | A_17_P27291274 | chr9:122159625-122159669 | 6.03E-03 | 2.32E-04 | 2.38 | Hypermethylated | Promoter |
| 189 | NDUFA9 | NADH dehydrogenase (ubiquinone) 1 alpha subcomplex, 9, 39kDa | A_17_P28595804 | chr12:4649142-4649186 | 5.61E-03 | 2.05E-04 | 1.71 | Hypermethylated | Promoter |
| 190 | NDUFAF1 | NADH dehydrogenase (ubiquinone) 1 alpha subcomplex, assembly factor 1 | A_17_P16782354 | chr15:41402289-41402333 | 3.27E-03 | 8.17E-05 | 1.86 | Hypermethylated | Promoter |
| 191 | NDUFAF2 | NADH dehydrogenase (ubiquinone) 1 alpha subcomplex, assembly factor 2 | A_17_P15694715 | chr5:60945335-60945385 | 8.15E-04 | 6.15E-06 | 2.53 | Hypermethylated | Promoter |
| 192 | NDUFB1 | NADH dehydrogenase (ubiquinone) 1 beta subcomplex, 1, 7kDa | A_17_P09475079 | chr14:92121460-92121504 | 2.40E-03 | 4.67E-05 | 2.62 | Hypermethylated | Promoter |
| 193 | NDUFB2 | NADH dehydrogenase (ubiquinone) 1 beta subcomplex, 2, 8kDa | A_17_P05828449 | chr7:140696624-140696668 | 9.45E-03 | 4.99E-04 | 2.35 | Hypermethylated | Promoter |
| 194 | NDUFB5 | NADH dehydrogenase (ubiquinone) 1 beta subcomplex, 5, 16kDa | A_17_P02804086 | chr3:179604714-179604761 | 3.54E-03 | 9.40E-05 | 2.28 | Hypermethylated | Promoter |
| 195 | NDUFB5 | NADH dehydrogenase (ubiquinone) 1 beta subcomplex, 5, 16kDa | A_17_P02804087 | chr3:179604888-179604932 | 3.72E-04 | 1.30E-06 | 3.07 | Hypermethylated | Promoter |
| 196 | NDUFB7 | NADH dehydrogenase (ubiquinone) 1 beta subcomplex, 7, 18kDa | A_17_P17112568 | chr19:14572094-14572140 | 9.11E-04 | 7.64E-06 | 2.56 | Hypermethylated | Promoter |
| 197 | NDUFS1 | NADH dehydrogenase (ubiquinone) Fe-S protein 1, 75kDa (NADH-coenzyme Q reductase) | A_17_P01836963 | chr2:206159479-206159525 | 7.34E-03 | 3.27E-04 | 1.86 | Hypermethylated | Promoter |
| 198 | NDUFS2 | NADH dehydrogenase (ubiquinone) Fe-S protein 2, 49kDa (NADH-coenzyme Q reductase) | A_17_P20607725 | chr1:161202507-161202551 | 3.74E-03 | 1.04E-04 | 1.72 | Hypermethylated | Promoter |
| 199 | NDUFV2 | NADH dehydrogenase (ubiquinone) flavoprotein 2, 24kDa | A_17_P31130411 | chr18:9102710-9102754 | 4.18E-03 | 1.26E-04 | 1.91 | Hypermethylated | Promoter |
| 200 | NDUFV3 | NADH dehydrogenase (ubiquinone) flavoprotein 3, 10kDa | A_17_P17239544 | chr21:42893169-42893213 | 2.22E-03 | 4.07E-05 | 2.54 | Hypomethylated | Promoter |
| 201 | NEUROD1 | neurogenic differentiation 1 | A_17_P15362114 | chr2:181680479-181680523 | 1.80E-03 | 2.79E-05 | 1.93 | Hypermethylated | Inside/exonic |
| 202 | NFATC1 | nuclear factor of activated T-cells, cytoplasmic, calcineurin-dependent 1 | A_17_P10846079 | chr18:79393516-79393560 | 6.03E-03 | 2.32E-04 | 1.97 | Hypomethylated | Promoter |
| 203 | NFATC1 | nuclear factor of activated T-cells, cytoplasmic, calcineurin-dependent 1 | A_17_P10846094 | chr18:79397697-79397750 | 4.71E-03 | 1.53E-04 | 2.20 | Hypomethylated | Promoter |
| 204 | NFATC1 | nuclear factor of activated T-cells, cytoplasmic, calcineurin-dependent 1 | A_17_P31449866 | chr18:79398023-79398067 | 5.67E-03 | 2.09E-04 | 2.41 | Hypomethylated | Promoter |
| 205 | NFATC1 | nuclear factor of activated T-cells, cytoplasmic, calcineurin-dependent 1 | A_17_P17088518 | chr18:79420431-79420475 | 4.84E-03 | 1.62E-04 | 2.95 | Hypomethylated | Transcription factor binding site |
| 206 | NFATC1 | nuclear factor of activated T-cells, cytoplasmic, calcineurin-dependent 1 | A_17_P10846231 | chr18:79420499-79420543 | 1.24E-03 | 1.40E-05 | 2.33 | Hypomethylated | Inside/intronic |
| 207 | NFATC1 | nuclear factor of activated T-cells, cytoplasmic, calcineurin-dependent 1 | A_17_P31450024 | chr18:79420636-79420680 | 4.49E-03 | 1.42E-04 | 2.45 | Hypomethylated | Inside/intronic |
| 208 | NFATC1 | nuclear factor of activated T-cells, cytoplasmic, calcineurin-dependent 1 | A_17_P10846232 | chr18:79420677-79420721 | 5.30E-04 | 2.62E-06 | 2.46 | Hypomethylated | Inside/intronic |
| 209 | NFATC1 | nuclear factor of activated T-cells, cytoplasmic, calcineurin-dependent 1 | A_17_P17088520 | chr18:79420759-79420803 | 6.05E-04 | 3.40E-06 | 2.40 | Hypomethylated | Inside/intronic |
| 210 | NFATC1 | nuclear factor of activated T-cells, cytoplasmic, calcineurin-dependent 1 | A_17_P10846543 | chr18:79467510-79467554 | 4.01E-04 | 1.51E-06 | 2.11 | Hypomethylated | Inside/intronic |
| 211 | NFATC1 | nuclear factor of activated T-cells, cytoplasmic, calcineurin-dependent 1 | A_17_P31450450 | chr18:79473428-79473472 | 5.84E-03 | 2.20E-04 | 3.73 | Hypomethylated | Inside/intronic |
| 212 | NFATC1 | nuclear factor of activated T-cells, cytoplasmic, calcineurin-dependent 1 | A_17_P17088690 | chr18:79473497-79473541 | 5.28E-03 | 1.86E-04 | 4.93 | Hypomethylated | Inside/intronic |
| 213 | NFATC1 | nuclear factor of activated T-cells, cytoplasmic, calcineurin-dependent 1 | A_17_P17088691 | chr18:79473586-79473631 | 3.06E-04 | 8.34E-07 | 6.99 | Hypomethylated | Inside/intronic |
| 214 | NFATC1 | nuclear factor of activated T-cells, cytoplasmic, calcineurin-dependent 1 | A_17_P31450453 | chr18:79473811-79473857 | 1.90E-03 | 3.07E-05 | 3.80 | Hypomethylated | Inside/intronic |
| 215 | NFATC1 | nuclear factor of activated T-cells, cytoplasmic, calcineurin-dependent 1 | A_17_P31450454 | chr18:79473912-79473956 | 1.87E-03 | 2.98E-05 | 5.09 | Hypomethylated | Inside/intronic |
| 216 | NFATC1 | nuclear factor of activated T-cells, cytoplasmic, calcineurin-dependent 1 | A_17_P17088697 | chr18:79474101-79474147 | 1.59E-03 | 2.20E-05 | 6.25 | Hypomethylated | Inside/intronic |
| 217 | NFATC1 | nuclear factor of activated T-cells, cytoplasmic, calcineurin-dependent 1 | A_17_P17088698 | chr18:79474183-79474228 | 3.67E-03 | 1.00E-04 | 5.41 | Hypomethylated | Inside/intronic |
| 218 | NFATC1 | nuclear factor of activated T-cells, cytoplasmic, calcineurin-dependent 1 | A_17_P17088699 | chr18:79474264-79474310 | 2.20E-04 | 3.76E-07 | 6.66 | Hypomethylated | Inside/intronic |
| 219 | NFATC1 | nuclear factor of activated T-cells, cytoplasmic, calcineurin-dependent 1 | A_17_P31450459 | chr18:79474387-79474432 | 2.78E-05 | 3.72E-10 | 4.14 | Hypomethylated | Inside/intronic |
| 220 | NFATC1 | nuclear factor of activated T-cells, cytoplasmic, calcineurin-dependent 1 | A_17_P31450460 | chr18:79474515-79474562 | 1.21E-03 | 1.31E-05 | 3.76 | Hypomethylated | Inside/intronic |
| 221 | NFATC1 | nuclear factor of activated T-cells, cytoplasmic, calcineurin-dependent 1 | A_17_P17088704 | chr18:79474723-79474767 | 6.65E-03 | 2.75E-04 | 4.32 | Hypomethylated | Inside/intronic |
| 222 | NFATC1 | nuclear factor of activated T-cells, cytoplasmic, calcineurin-dependent 1 | A_17_P17088705 | chr18:79474788-79474832 | 2.15E-04 | 3.40E-07 | 3.06 | Hypomethylated | Inside/intronic |
| 223 | NFATC1 | nuclear factor of activated T-cells, cytoplasmic, calcineurin-dependent 1 | A_17_P31450464 | chr18:79475043-79475088 | 4.17E-03 | 1.25E-04 | 4.79 | Hypomethylated | Inside/intronic |
| 224 | NFATC1 | nuclear factor of activated T-cells, cytoplasmic, calcineurin-dependent 1 | A_17_P17088708 | chr18:79475127-79475177 | 3.49E-03 | 9.18E-05 | 4.23 | Hypomethylated | Inside/intronic |
| 225 | NFATC1 | nuclear factor of activated T-cells, cytoplasmic, calcineurin-dependent 1 | A_17_P17088709 | chr18:79475217-79475261 | 9.21E-04 | 7.85E-06 | 4.75 | Hypomethylated | Inside/intronic |
| 226 | NFATC1 | nuclear factor of activated T-cells, cytoplasmic, calcineurin-dependent 1 | A_17_P17088710 | chr18:79475303-79475347 | 1.21E-03 | 1.34E-05 | 5.65 | Hypomethylated | Inside/intronic |
| 227 | NFATC1 | nuclear factor of activated T-cells, cytoplasmic, calcineurin-dependent 1 | A_17_P31450468 | chr18:79475417-79475461 | 9.52E-04 | 8.40E-06 | 5.98 | Hypomethylated | Inside/intronic |
| 228 | NFATC1 | nuclear factor of activated T-cells, cytoplasmic, calcineurin-dependent 1 | A_17_P17088714 | chr18:79475590-79475634 | 3.66E-04 | 1.23E-06 | 6.11 | Hypomethylated | Inside/intronic |
| 229 | NFATC1 | nuclear factor of activated T-cells, cytoplasmic, calcineurin-dependent 1 | A_17_P31450470 | chr18:79475647-79475693 | 6.39E-03 | 2.55E-04 | 1.56 | Hypomethylated | Inside/intronic |
| 230 | NFATC1 | nuclear factor of activated T-cells, cytoplasmic, calcineurin-dependent 1 | A_17_P17088717 | chr18:79476148-79476192 | 1.21E-03 | 1.33E-05 | 1.86 | Hypomethylated | Inside/intronic |
| 231 | NFATC1 | nuclear factor of activated T-cells, cytoplasmic, calcineurin-dependent 1 | A_17_P17088752 | chr18:79483391-79483435 | 5.26E-03 | 1.85E-04 | 2.86 | Hypomethylated | Inside/intronic |
| 232 | NFATC1 | nuclear factor of activated T-cells, cytoplasmic, calcineurin-dependent 1 | A_17_P31450618 | chr18:79494287-79494331 | 2.71E-03 | 5.78E-05 | 4.12 | Hypomethylated | Inside/intronic |
| 233 | NFATC1 | nuclear factor of activated T-cells, cytoplasmic, calcineurin-dependent 1 | A_17_P17088799 | chr18:79494888-79494932 | 2.75E-03 | 5.93E-05 | 4.87 | Hypomethylated | Inside/intronic |
| 234 | NFATC1 | nuclear factor of activated T-cells, cytoplasmic, calcineurin-dependent 1 | A_17_P10846714 | chr18:79495723-79495767 | 5.48E-03 | 1.97E-04 | 3.42 | Hypomethylated | Inside/intronic |
| 235 | NFATC1 | nuclear factor of activated T-cells, cytoplasmic, calcineurin-dependent 1 | A_17_P10846715 | chr18:79495813-79495859 | 3.34E-03 | 8.45E-05 | 2.72 | Hypomethylated | Promoter flanking region |
| 236 | NFATC1 | nuclear factor of activated T-cells, cytoplasmic, calcineurin-dependent 1 | A_17_P17088802 | chr18:79495864-79495908 | 6.24E-04 | 3.56E-06 | 2.81 | Hypomethylated | Promoter flanking region |
| 237 | NFATC1 | nuclear factor of activated T-cells, cytoplasmic, calcineurin-dependent 1 | A_17_P10846716 | chr18:79496052-79496107 | 9.95E-05 | 3.23E-08 | 3.99 | Hypomethylated | Promoter flanking region |
| 238 | NFATC1 | nuclear factor of activated T-cells, cytoplasmic, calcineurin-dependent 1 | A_17_P10846765 | chr18:79511071-79511123 | 5.48E-03 | 1.97E-04 | 2.05 | Hypomethylated | Inside/intronic |
| 239 | NFATC1 | nuclear factor of activated T-cells, cytoplasmic, calcineurin-dependent 1 | A_17_P17088851 | chr18:79524436-79524480 | 5.28E-03 | 1.86E-04 | 1.90 | Hypermethylated | Promoter |
| 240 | NFATC1 | nuclear factor of activated T-cells, cytoplasmic, calcineurin-dependent 1 | A_17_P17088857 | chr18:79525049-79525093 | 1.09E-03 | 1.07E-05 | 1.73 | Hypomethylated | Inside/intronic |
| 241 | NFATC1 | nuclear factor of activated T-cells, cytoplasmic, calcineurin-dependent 1 | A_17_P17088860 | chr18:79525280-79525324 | 1.44E-03 | 1.84E-05 | 1.80 | Hypomethylated | Inside/intronic |
| 242 | NFATC1 | nuclear factor of activated T-cells, cytoplasmic, calcineurin-dependent 1 | A_17_P31450797 | chr18:79525445-79525489 | 2.19E-03 | 3.97E-05 | 1.61 | Hypomethylated | Inside/intronic |
| 243 | NFATC1 | nuclear factor of activated T-cells, cytoplasmic, calcineurin-dependent 1 | A_17_P10846855 | chr18:79525531-79525575 | 2.57E-04 | 5.24E-07 | 2.10 | Hypomethylated | Inside/intronic |
| 244 | NFATC1 | nuclear factor of activated T-cells, cytoplasmic, calcineurin-dependent 1 | A_17_P10846892 | chr18:79528997-79529041 | 1.26E-03 | 1.44E-05 | 2.12 | Hypomethylated | Transcription factor binding site/CTCF binding site |
| 245 | NFATC1 | nuclear factor of activated T-cells, cytoplasmic, calcineurin-dependent 1 | A_17_P31450831 | chr18:79529077-79529123 | 7.95E-05 | 1.93E-08 | 3.79 | Hypomethylated | Transcription factor binding site/CTCF binding site |
| 246 | NFATC1 | nuclear factor of activated T-cells, cytoplasmic, calcineurin-dependent 1 | A_17_P31450832 | chr18:79529172-79529216 | 9.16E-03 | 4.73E-04 | 2.09 | Hypomethylated | Transcription factor binding site/CTCF binding site |
| 247 | NFATC1 | nuclear factor of activated T-cells, cytoplasmic, calcineurin-dependent 1 | A_17_P10846895 | chr18:79529267-79529326 | 8.42E-04 | 6.66E-06 | 2.12 | Hypomethylated | CTCF binding site |
| 248 | NKX2-2 | NK2 homeobox 2 | A_17_P11110593 | chr20:21510983-21511038 | 6.74E-04 | 4.23E-06 | 3.95 | Hypermethylated | Promoter |
| 249 | NKX2-2 | NK2 homeobox 2 | A_17_P11110595 | chr20:21511309-21511353 | 5.85E-03 | 2.20E-04 | 3.29 | Hypomethylated | Inside/exonic |
| 250 | NKX2-2 | NK2 homeobox 2 | A_17_P31754608 | chr20:21513238-21513290 | 7.78E-04 | 5.60E-06 | 8.97 | Hypermethylated | Promoter |
| 251 | NR3C1 | nuclear receptor subfamily 3, group C, member 1 (glucocorticoid receptor) | A_17_P04337419 | chr5:143402560-143402613 | 4.79E-03 | 1.58E-04 | 2.29 | Hypermethylated | Promoter |
| 252 | NR3C1 | nuclear receptor subfamily 3, group C, member 1 (glucocorticoid receptor) | A_17_P15748741 | chr5:143404454-143404498 | 8.76E-03 | 4.38E-04 | 1.61 | Hypomethylated | Promoter |
| 253 | PDE3A | phosphodiesterase 3A, cGMP-inhibited | A_17_P28664438 | chr12:20369249-20369293 | 1.41E-04 | 1.08E-07 | 2.41 | Hypomethylated | Promoter |
| 254 | PDE3A | phosphodiesterase 3A, cGMP-inhibited | A_17_P08231879 | chr12:20370105-20370149 | 6.11E-03 | 2.37E-04 | 2.94 | Hypomethylated | Promoter |
| 255 | PDE3A | phosphodiesterase 3A, cGMP-inhibited | A_17_P16539240 | chr12:20551961-20552005 | 2.69E-03 | 5.68E-05 | 1.99 | Hypomethylated | Inside/intronic |
| 256 | PDE3A | phosphodiesterase 3A, cGMP-inhibited | A_17_P16539241 | chr12:20552229-20552273 | 6.93E-03 | 2.95E-04 | 1.53 | Hypomethylated | Inside/intronic |
| 257 | PDE3A | phosphodiesterase 3A, cGMP-inhibited | A_17_P16539247 | chr12:20552943-20552987 | 3.71E-03 | 1.02E-04 | 1.81 | Hypomethylated | Inside/intronic |
| 258 | PDGFA | platelet-derived growth factor alpha polypeptide | A_17_P32888010 | chr7:518065-518109 | 7.30E-03 | 3.24E-04 | 1.84 | Hypomethylated | Promoter |
| 259 | PDGFA | platelet-derived growth factor alpha polypeptide | A_17_P25470204 | chr7:519055-519099 | 9.95E-03 | 5.45E-04 | 2.48 | Hypomethylated | Promoter |
| 260 | PDGFRA | platelet-derived growth factor receptor, alpha polypeptide | A_17_P03104446 | chr4:54226140-54226184 | 3.19E-03 | 7.77E-05 | 1.64 | Hypomethylated | Inside/intronic |
| 261 | PDGFRA | platelet-derived growth factor receptor, alpha polypeptide | A_17_P23258814 | chr4:54226744-54226788 | 4.04E-03 | 1.19E-04 | 2.15 | Hypermethylated | Inside/intronic |
| 262 | PDGFRA | platelet-derived growth factor receptor, alpha polypeptide | A_17_P03104480 | chr4:54230302-54230350 | 4.42E-03 | 1.38E-04 | 2.01 | Hypermethylated | Promoter |
| 263 | PDK1 | pyruvate dehydrogenase kinase, isozyme 1 | A_17_P21801710 | chr2:172555724-172555768 | 1.66E-03 | 2.39E-05 | 2.24 | Hypermethylated | Promoter |
| 264 | PDK1 | pyruvate dehydrogenase kinase, isozyme 1 | A_17_P21801717 | chr2:172556468-172556512 | 8.10E-03 | 3.86E-04 | 1.53 | Hypermethylated | Promoter |
| 265 | PDK2 | pyruvate dehydrogenase kinase, isozyme 2 | A_17_P16994657 | chr17:50095376-50095420 | 2.20E-04 | 3.79E-07 | 3.64 | Hypomethylated | Promoter |
| 266 | PDK2 | pyruvate dehydrogenase kinase, isozyme 2 | A_17_P16994681 | chr17:50105885-50105929 | 5.04E-03 | 1.73E-04 | 1.67 | Hypomethylated | Inside/exonic |
| 267 | PDX1 | pancreatic and duodenal homeobox 1 | A_17_P29187513 | chr13:27921515-27921560 | 6.55E-03 | 2.67E-04 | 2.81 | Hypermethylated | Promoter |
| 268 | PFKL | phosphofructokinase, liver | A_17_P32073720 | chr21:44303955-44304014 | 1.65E-04 | 1.71E-07 | 3.64 | Hypomethylated | Inside/intronic |
| 269 | PFKM | phosphofructokinase, muscle | A_17_P28771148 | chr12:48106234-48106289 | 1.10E-03 | 1.08E-05 | 3.55 | Hypermethylated | Promoter |
| 270 | PFKP | phosphofructokinase, platelet | A_17_P07031578 | chr10:3068706-3068750 | 5.02E-03 | 1.71E-04 | 1.90 | Hypomethylated | Promoter |
| 271 | PFKP | phosphofructokinase, platelet | A_17_P16285735 | chr10:3068797-3068841 | 8.36E-03 | 4.07E-04 | 2.90 | Hypomethylated | Promoter |
| 272 | PPARA | peroxisome proliferator-activated receptor alpha | A_17_P17292235 | chr22:46149854-46149898 | 3.07E-04 | 8.61E-07 | 2.48 | Hypermethylated | Promoter |
| 273 | PPARA | peroxisome proliferator-activated receptor alpha | A_17_P11530608 | chr22:46151220-46151265 | 1.23E-03 | 1.37E-05 | 2.84 | Hypermethylated | Promoter |
| 274 | PPARA | peroxisome proliferator-activated receptor alpha | A_17_P32219860 | chr22:46214155-46214200 | 6.62E-03 | 2.72E-04 | 1.87 | Hypomethylated | Inside/intronic |
| 275 | PPARGC1B | peroxisome proliferator-activated receptor gamma, coactivator 1 beta | A_17_P15751799 | chr5:149731042-149731086 | 2.58E-03 | 5.31E-05 | 1.78 | Hypermethylated | Promoter |
| 276 | PPARGC1B | peroxisome proliferator-activated receptor gamma, coactivator 1 beta | A_17_P04365633 | chr5:149824746-149824790 | 5.41E-03 | 1.93E-04 | 1.71 | Hypomethylated | Promoter flanking region |
| 277 | PPRC1 | peroxisome proliferator-activated receptor gamma, coactivator-related 1 | A_17_P27827903 | chr10:102133585-102133629 | 6.38E-03 | 2.55E-04 | 1.56 | Hypermethylated | Promoter |
| 278 | PRDM16 | PR domain containing 16 | A_17_P15013834 | chr1:3073705-3073749 | 3.89E-03 | 1.12E-04 | 1.92 | Hypermethylated | Promoter |
| 279 | PRDM16 | PR domain containing 16 | A_17_P15014136 | chr1:3186373-3186417 | 9.97E-03 | 5.47E-04 | 1.42 | Hypomethylated | Promoter Flanking region |
| 280 | PRDM16 | PR domain containing 16 | A_17_P20016372 | chr1:3188530-3188574 | 8.91E-03 | 4.51E-04 | 2.04 | Hypomethylated | Promoter Flanking region |
| 281 | PRDM16 | PR domain containing 16 | A_17_P00008696 | chr1:3225598-3225655 | 4.23E-03 | 1.29E-04 | 2.69 | Hypomethylated | Inside/intronic |
| 282 | PRDM16 | PR domain containing 16 | A_17_P00009554 | chr1:3358416-3358460 | 9.18E-03 | 4.75E-04 | 1.90 | Hypomethylated | Inside/intronic |
| 283 | PRDM16 | PR domain containing 16 | A_17_P00009555 | chr1:3358498-3358542 | 3.04E-03 | 7.07E-05 | 2.54 | Hypomethylated | Inside/intronic |
| 284 | PRDM16 | PR domain containing 16 | A_17_P00009945 | chr1:3412512-3412556 | 5.66E-03 | 2.08E-04 | 1.91 | Hypomethylated | Inside/exonic |
| 285 | PRDM16 | PR domain containing 16 | A_17_P20018045 | chr1:3412582-3412626 | 8.82E-03 | 4.45E-04 | 2.34 | Hypomethylated | Inside/exonic |
| 286 | PRDM16 | PR domain containing 16 | A_17_P15014680 | chr1:3412664-3412708 | 4.11E-03 | 1.23E-04 | 2.62 | Hypomethylated | Inside/exonic |
| 287 | PRKAA1 | protein kinase, AMP-activated, alpha 1 catalytic subunit | A_17_P24064865 | chr5:40797928-40797972 | 6.32E-03 | 2.51E-04 | 1.71 | Hypomethylated | Promoter |
| 288 | PRKAB2 | protein kinase, AMP-activated, beta 2 non-catalytic subunit | A_17_P15118205 | chr1:147172538-147172582 | 1.36E-03 | 1.65E-05 | 2.05 | Hypomethylated | Promoter |
| 289 | PRKACG | protein kinase, cAMP-dependent, catalytic, gamma | A_17_P06716069 | chr9:69013652-69013696 | 6.63E-03 | 2.74E-04 | 1.75 | Hypomethylated | Inside/exonic |
| 290 | PRKAR1A | protein kinase, cAMP-dependent, regulatory, type I, alpha (tissue specific extinguisher 1) | A_17_P17011312 | chr17:68512618-68512662 | 2.94E-03 | 6.68E-05 | 2.55 | Hypomethylated | Promoter |
| 291 | PRKAR2B | protein kinase, cAMP-dependent, regulatory, type II, beta | A_17_P25928966 | chr7:107044995-107045039 | 4.81E-03 | 1.59E-04 | 1.98 | Hypermethylated | Promoter |
| 292 | PRKCB | protein kinase C, beta | A_17_P16869685 | chr16:23836685-23836729 | 8.14E-03 | 3.89E-04 | 2.26 | Hypermethylated | Promoter |
| 293 | PRKCE | protein kinase C, epsilon | A_17_P15239539 | chr2:45650775-45650819 | 2.94E-03 | 6.67E-05 | 1.90 | Hypomethylated | Promoter |
| 294 | PRKCH | protein kinase C, eta | A_17_P09333615 | chr14:61321305-61321350 | 4.66E-03 | 1.51E-04 | 1.61 | Hypermethylated | Promoter |
| 295 | PRKCI | protein kinase C, iota | A_17_P15510358 | chr3:170222827-170222871 | 7.99E-03 | 3.77E-04 | 1.63 | Hypermethylated | Promoter |
| 296 | PRKCZ | protein kinase C, zeta | A_17_P15010760 | chr1:2074063-2074107 | 1.96E-03 | 3.24E-05 | 1.84 | Hypermethylated | Promoter |
| 297 | PRKCZ | protein kinase C, zeta | A_17_P00003445 | chr1:2132560-2132604 | 7.18E-03 | 3.15E-04 | 1.88 | Hypomethylated | Promoter/ CTCF binding site |
| 298 | PRKCZ | protein kinase C, zeta | A_17_P15010948 | chr1:2150847-2150891 | 3.90E-03 | 1.12E-04 | 1.95 | Hypomethylated | Inside/exonic |
| 299 | PRKCZ | protein kinase C, zeta | A_17_P20009863 | chr1:2150913-2150966 | 9.55E-03 | 5.08E-04 | 1.68 | Hypomethylated | Inside/exonic |
| 300 | PTPRN2 | protein tyrosine phosphatase, receptor type, N polypeptide 2 | A_17_P16047205 | chr7:157613708-157613752 | 8.73E-03 | 4.35E-04 | 1.66 | Hypomethylated | Promoter |
| 301 | PTPRN2 | protein tyrosine phosphatase, receptor type, N polypeptide 2 | A_17_P16047212 | chr7:157617188-157617232 | 2.53E-04 | 5.02E-07 | 2.74 | Hypomethylated | Inside/intronic |
| 302 | PTPRN2 | protein tyrosine phosphatase, receptor type, N polypeptide 2 | A_17_P16047282 | chr7:157646983-157647027 | 2.42E-03 | 4.73E-05 | 7.19 | Hypomethylated | Inside/intronic |
| 303 | PTPRN2 | protein tyrosine phosphatase, receptor type, N polypeptide 2 | A_17_P26168003 | chr7:157647075-157647119 | 2.03E-04 | 2.85E-07 | 4.66 | Hypomethylated | Inside/intronic |
| 304 | PTPRN2 | protein tyrosine phosphatase, receptor type, N polypeptide 2 | A_17_P16047316 | chr7:157647561-157647605 | 7.12E-04 | 4.77E-06 | 6.51 | Hypomethylated | Inside/intronic |
| 305 | PTPRN2 | protein tyrosine phosphatase, receptor type, N polypeptide 2 | A_17_P16047290 | chr7:157647721-157647765 | 7.95E-05 | 2.28E-08 | 5.90 | Hypomethylated | Inside/intronic |
| 306 | PTPRN2 | protein tyrosine phosphatase, receptor type, N polypeptide 2 | A_17_P16047300 | chr7:157648537-157648581 | 8.65E-03 | 4.29E-04 | 6.29 | Hypomethylated | Inside/intronic |
| 307 | PTPRN2 | protein tyrosine phosphatase, receptor type, N polypeptide 2 | A_17_P16047310 | chr7:157649431-157649475 | 9.29E-03 | 4.85E-04 | 5.52 | Hypomethylated | Inside/intronic |
| 308 | PTPRN2 | protein tyrosine phosphatase, receptor type, N polypeptide 2 | A_17_P16047312 | chr7:157649591-157649635 | 3.89E-03 | 1.12E-04 | 4.09 | Hypomethylated | Inside/intronic |
| 309 | PTPRN2 | protein tyrosine phosphatase, receptor type, N polypeptide 2 | A_17_P05898618 | chr7:157650000-157650044 | 1.31E-04 | 8.21E-08 | 4.04 | Hypomethylated | Inside/intronic |
| 310 | PTPRN2 | protein tyrosine phosphatase, receptor type, N polypeptide 2 | A_17_P05898861 | chr7:157684104-157684159 | 9.99E-03 | 5.49E-04 | 2.19 | Hypermethylated | Inside/intronic |
| 311 | PTPRN2 | protein tyrosine phosphatase, receptor type, N polypeptide 2 | A_17_P05898863 | chr7:157684621-157684665 | 8.08E-03 | 3.84E-04 | 1.93 | Hypomethylated | Inside/intronic |
| 312 | PTPRN2 | protein tyrosine phosphatase, receptor type, N polypeptide 2 | A_17_P16047406 | chr7:157685291-157685335 | 8.74E-03 | 4.37E-04 | 2.78 | Hypomethylated | Inside/intronic |
| 313 | PTPRN2 | protein tyrosine phosphatase, receptor type, N polypeptide 2 | A_17_P26168443 | chr7:157701733-157701777 | 2.32E-03 | 4.37E-05 | 2.41 | Hypomethylated | Inside/intronic |
| 314 | PTPRN2 | protein tyrosine phosphatase, receptor type, N polypeptide 2 | A_17_P16048717 | chr7:158245362-158245406 | 1.30E-04 | 8.07E-08 | 2.34 | Hypomethylated | Inside/intronic |
| 315 | PTPRN2 | protein tyrosine phosphatase, receptor type, N polypeptide 2 | A_17_P16048769 | chr7:158266077-158266122 | 1.30E-04 | 7.74E-08 | 2.48 | Hypermethylated | Inside/intronic |
| 316 | RARA | retinoic acid receptor, alpha | A_17_P16979962 | chr17:40316834-40316878 | 2.87E-03 | 6.41E-05 | 1.63 | Hypomethylated | Promoter |
| 317 | RARA | retinoic acid receptor, alpha | A_17_P30888980 | chr17:40317938-40317982 | 6.63E-03 | 2.73E-04 | 1.91 | Hypermethylated | Promoter |
| 318 | RARA | retinoic acid receptor, alpha | A_17_P10318218 | chr17:40317970-40318019 | 5.16E-03 | 1.79E-04 | 5.47 | Hypermethylated | Promoter |
| 319 | RARA | retinoic acid receptor, alpha | A_17_P16979969 | chr17:40318428-40318472 | 9.59E-03 | 5.13E-04 | 1.60 | Hypermethylated | Promoter |
| 320 | RARA | retinoic acid receptor, alpha | A_17_P30889131 | chr17:40341795-40341839 | 9.92E-03 | 5.42E-04 | 1.41 | Hypermethylated | Promoter |
| 321 | RORA | RAR-related orphan receptor A | A_17_P16796011 | chr15:61229491-61229535 | 5.46E-03 | 1.96E-04 | 2.09 | Hypermethylated | Promoter/ CTCF binding site |
| 322 | RORA | RAR-related orphan receptor A | A_17_P30191805 | chr15:61229558-61229602 | 6.17E-03 | 2.40E-04 | 3.15 | Hypermethylated | Promoter/ CTCF binding site |
| 323 | RORA | RAR-related orphan receptor A | A_17_P16796012 | chr15:61229664-61229713 | 5.56E-03 | 2.02E-04 | 2.44 | Hypermethylated | Promoter/ CTCF binding site |
| 324 | RORB | RAR-related orphan receptor B | A_17_P06741104 | chr9:74498431-74498475 | 2.02E-03 | 3.42E-05 | 1.90 | Hypermethylated | Promoter |
| 325 | RPTOR | regulatory associated protein of MTOR, complex 1 | A_17_P17024200 | chr17:80727136-80727180 | 3.13E-03 | 7.49E-05 | 1.96 | Hypomethylated | Promoter flanking region/ CTCF binding site |
| 326 | RPTOR | regulatory associated protein of MTOR, complex 1 | A_17_P31076648 | chr17:80815719-80815774 | 4.43E-03 | 1.39E-04 | 1.68 | Hypomethylated | Inside/intronic |
| 327 | RPTOR | regulatory associated protein of MTOR, complex 1 | A_17_P17024320 | chr17:80815917-80815961 | 5.37E-03 | 1.91E-04 | 1.85 | Hypomethylated | Enhancer |
| 328 | RPTOR | regulatory associated protein of MTOR, complex 1 | A_17_P10490579 | chr17:80889713-80889757 | 8.30E-03 | 4.02E-04 | 1.67 | Hypomethylated | Inside/intronic |
| 329 | RPTOR | regulatory associated protein of MTOR, complex 1 | A_17_P31077129 | chr17:80890047-80890091 | 8.48E-03 | 4.17E-04 | 1.67 | Hypomethylated | Inside/intronic |
| 330 | RPTOR | regulatory associated protein of MTOR, complex 1 | A_17_P17024477 | chr17:80890811-80890855 | 6.05E-04 | 3.39E-06 | 2.20 | Hypomethylated | Inside/intronic |
| 331 | RPTOR | regulatory associated protein of MTOR, complex 1 | A_17_P10490586 | chr17:80890916-80890960 | 2.53E-03 | 5.11E-05 | 2.39 | Hypomethylated | Inside/intronic |
| 332 | RPTOR | regulatory associated protein of MTOR, complex 1 | A_17_P17024489 | chr17:80896597-80896641 | 9.95E-04 | 9.01E-06 | 2.74 | Hypomethylated | Inside/intronic |
| 333 | RPTOR | regulatory associated protein of MTOR, complex 1 | A_17_P31077231 | chr17:80906217-80906261 | 7.82E-04 | 5.67E-06 | 2.15 | Hypomethylated | Inside/intronic |
| 334 | SCD | stearoyl-CoA desaturase (delta-9-desaturase) | A_17_P16377646 | chr10:100347232-100347276 | 4.62E-03 | 1.49E-04 | 2.04 | Hypermethylated | Promoter |
| 335 | SLC27A1 | solute carrier family 27 (fatty acid transporter), member 1 | A_17_P17115747 | chr19:17469543-17469587 | 3.60E-03 | 9.69E-05 | 1.51 | Hypomethylated | Promoter |
| 336 | SLC2A11 | solute carrier family 2 (facilitated glucose transporter), member 11 | A_17_P11444408 | chr22:23856978-23857023 | 9.52E-03 | 5.06E-04 | 2.81 | Hypermethylated | Promoter |
| 337 | SLC2A4 | solute carrier family 2 (facilitated glucose transporter), member 4 | A_17_P16940266 | chr17:7281629-7281673 | 1.87E-03 | 2.98E-05 | 1.90 | Hypermethylated | Promoter |
| 338 | SLC2A5 | solute carrier family 2 (facilitated glucose/fructose transporter), member 5 | A_17_P15021316 | chr1:9039241-9039285 | 6.42E-03 | 2.57E-04 | 1.60 | Hypermethylated | Inside/intronic |
| 339 | SLC2A5 | solute carrier family 2 (facilitated glucose/fructose transporter), member 5 | A_17_P15021341 | chr1:9071643-9071687 | 5.21E-03 | 1.82E-04 | 1.84 | Hypomethylated | CTCF binding site |
| 340 | SLC2A6 | solute carrier family 2 (facilitated glucose transporter), member 6 | A_17_P27342809 | chr9:133479420-133479464 | 2.86E-03 | 6.36E-05 | 1.81 | Hypomethylated | Promoter |
| 341 | SLC2A8 | solute carrier family 2 (facilitated glucose transporter), member 8 | A_17_P27315238 | chr9:127398013-127398057 | 8.62E-03 | 4.26E-04 | 1.88 | Hypermethylated | Promoter |
| 342 | SLC7A1 | solute carrier family 7 (cationic amino acid transporter, y+ system), member 1 | A_17_P16622549 | chr13:29594784-29594828 | 2.03E-03 | 3.45E-05 | 2.60 | Hypomethylated | Promoter |
| 343 | SOCS3 | suppressor of cytokine signaling 3 | A_17_P17020045 | chr17:78358712-78358756 | 8.58E-03 | 4.24E-04 | 2.48 | Hypomethylated | Promoter |
| 344 | SOCS3 | suppressor of cytokine signaling 3 | A_17_P17020047 | chr17:78359075-78359119 | 7.85E-03 | 3.67E-04 | 1.88 | Hypomethylated | Promoter |
| 345 | SOD1 | superoxide dismutase 1, soluble | A_17_P17230758 | chr21:31659980-31660024 | 2.03E-03 | 3.45E-05 | 1.74 | Hypomethylated | Promoter |
| 346 | STARD4 | StAR-related lipid transfer (START) domain containing 4 | A_17_P04191823 | chr5:111512256-111512300 | 5.16E-03 | 1.79E-04 | 1.95 | Hypomethylated | Promoter |
| 347 | STARD9 | StAR-related lipid transfer (START) domain containing 9 | A_17_P09616380 | chr15:42576248-42576301 | 1.19E-03 | 1.28E-05 | 2.39 | Hypermethylated | Promoter |
| 348 | STARD9 | StAR-related lipid transfer (START) domain containing 9 | A_17_P16783301 | chr15:42576358-42576404 | 4.77E-03 | 1.57E-04 | 2.63 | Hypermethylated | Promoter |
| 349 | STARD9 | StAR-related lipid transfer (START) domain containing 9 | A_17_P30106706 | chr15:42581699-42581758 | 9.32E-03 | 4.87E-04 | 1.47 | Hypermethylated | Promoter |
| 350 | TBX1 | T-box 1 | A_17_P17255429 | chr22:19767438-19767482 | 7.30E-03 | 3.24E-04 | 1.72 | Hypermethylated | CTCF binding site |
| 351 | TBX5 | T-box 5 | A_17_P08627280 | chr12:114399569-114399620 | 9.74E-03 | 5.25E-04 | 2.19 | Hypermethylated | Inside/exonic |
| 352 | TBX5 | T-box 5 | A_17_P29062472 | chr12:114403096-114403140 | 5.54E-03 | 2.01E-04 | 2.04 | Hypermethylated | Promoter |
| 353 | TCF7L2 | transcription factor 7-like 2 (T-cell specific, HMG-box) | A_17_P07488727 | chr10:112950714-112950765 | 7.48E-03 | 3.37E-04 | 4.36 | Hypermethylated | Promoter |
| 354 | TCF7L2 | transcription factor 7-like 2 (T-cell specific, HMG-box) | A_17_P07490235 | chr10:113165685-113165729 | 7.70E-03 | 3.55E-04 | 1.47 | Hypermethylated | CTCF binding site |
| 355 | TFAP2A | transcription factor AP-2 alpha (activating enhancer binding protein 2 alpha) | A_17_P24738219 | chr6:10398416-10398463 | 2.92E-04 | 7.36E-07 | 2.04 | Hypermethylated | Enhancer |
| 356 | TFAP2A | transcription factor AP-2 alpha (activating enhancer binding protein 2 alpha) | A_17_P04550249 | chr6:10414145-10414189 | 6.16E-04 | 3.50E-06 | 2.01 | Hypermethylated | Promoter/CTCF binding site |
| 357 | TFAP2A | transcription factor AP-2 alpha (activating enhancer binding protein 2 alpha) | A_17_P15784720 | chr6:10415375-10415419 | 2.65E-03 | 5.57E-05 | 2.02 | Hypomethylated | Promoter/CTCF binding site |
| 358 | TFAP2A | transcription factor AP-2 alpha (activating enhancer binding protein 2 alpha) | A_17_P24738376 | chr6:10417226-10417270 | 5.93E-03 | 2.25E-04 | 2.29 | Hypermethylated | Promoter |
| 359 | TMEM26 | transmembrane protein 26 | A_17_P07263041 | chr10:61452758-61452803 | 8.98E-03 | 4.58E-04 | 2.06 | Hypermethylated | Promoter |
| 360 | VEGFA | vascular endothelial growth factor A | A_17_P15811303 | chr6:43772004-43772048 | 1.03E-03 | 9.57E-06 | 1.83 | Hypomethylated | Promoter |
| 361 | VLDLR | very low density lipoprotein receptor | A_17_P26838242 | chr9:2621969-2622013 | 9.01E-03 | 4.61E-04 | 1.59 | Hypermethylated | Promoter |
| 362 | WNT2B | wingless-type MMTV integration site family, member 2B | A_17_P15100562 | chr1:112508233-112508277 | 9.58E-03 | 5.11E-04 | 1.55 | Hypomethylated | Promoter |
| 363 | WNT2B | wingless-type MMTV integration site family, member 2B | A_17_P00463372 | chr1:112508657-112508701 | 9.71E-03 | 5.22E-04 | 1.81 | Hypomethylated | Promoter |
| 364 | WNT2B | wingless-type MMTV integration site family, member 2B | A_17_P00463375 | chr1:112509621-112509665 | 7.74E-04 | 5.53E-06 | 1.85 | Hypomethylated | Promoter |
| 365 | WNT3 | wingless-type MMTV integration site family, member 3 | A_17_P10342392 | chr17:46818658-46818710 | 1.10E-03 | 1.08E-05 | 2.98 | Hypermethylated | Promoter |
| 366 | WNT3A | wingless-type MMTV integration site family, member 3A | A_17_P00844036 | chr1:228007333-228007377 | 4.67E-03 | 1.51E-04 | 1.92 | Hypermethylated | Inside/intronic |
| 367 | WNT3A | wingless-type MMTV integration site family, member 3A | A_17_P00844040 | chr1:228008159-228008203 | 6.50E-03 | 2.64E-04 | 2.21 | Hypomethylated | Inside/intronic |
| 368 | WNT3A | wingless-type MMTV integration site family, member 3A | A_17_P20913971 | chr1:228008282-228008327 | 8.28E-03 | 4.00E-04 | 1.65 | Hypermethylated | Inside/intronic |
| 369 | WNT3A | wingless-type MMTV integration site family, member 3A | A_17_P00844182 | chr1:228037838-228037882 | 3.35E-03 | 8.51E-05 | 2.39 | Hypomethylated | Inside/intronic |
| 370 | WNT7A | wingless-type MMTV integration site family, member 7A | A_17_P22204062 | chr3:13879202-13879246 | 4.25E-03 | 1.30E-04 | 1.52 | Hypomethylated | Promoter |
| 371 | WNT7B | wingless-type MMTV integration site family, member 7B | A_17_P32218505 | chr22:45975947-45975991 | 3.47E-03 | 9.11E-05 | 2.46 | Hypomethylated | Promoter |
| 372 | WNT7B | wingless-type MMTV integration site family, member 7B | A_17_P32218508 | chr22:45976603-45976647 | 3.68E-04 | 1.25E-06 | 2.16 | Hypomethylated | Promoter |
| 373 | WNT9A | wingless-type MMTV integration site family, member 9A | A_17_P20913692 | chr1:227947124-227947168 | 2.95E-04 | 7.54E-07 | 3.38 | Hypomethylated | Promoter |
| 374 | WNT9A | wingless-type MMTV integration site family, member 9A | A_17_P15188008 | chr1:227947617-227947661 | 1.70E-03 | 2.52E-05 | 2.94 | Hypomethylated | Promoter |
| 375 | WNT9B | wingless-type MMTV integration site family, member 9B | A_17_P10342561 | chr17:46851036-46851080 | 3.24E-03 | 8.02E-05 | 1.86 | Hypomethylated | Inside/intronic |

Only probes described in the manuscript are shown.
