# Supplementary figures and images for: Epigenetic Regulation of Processes Related to High Level of Fibroblast Growth Factor 21 in Obese Subjects
Source: Genes (Basel). 2021 Feb 21;12(2):307. doi: 10.3390/genes12020307 (PMC7926457; doi:10.3390/genes12020307)

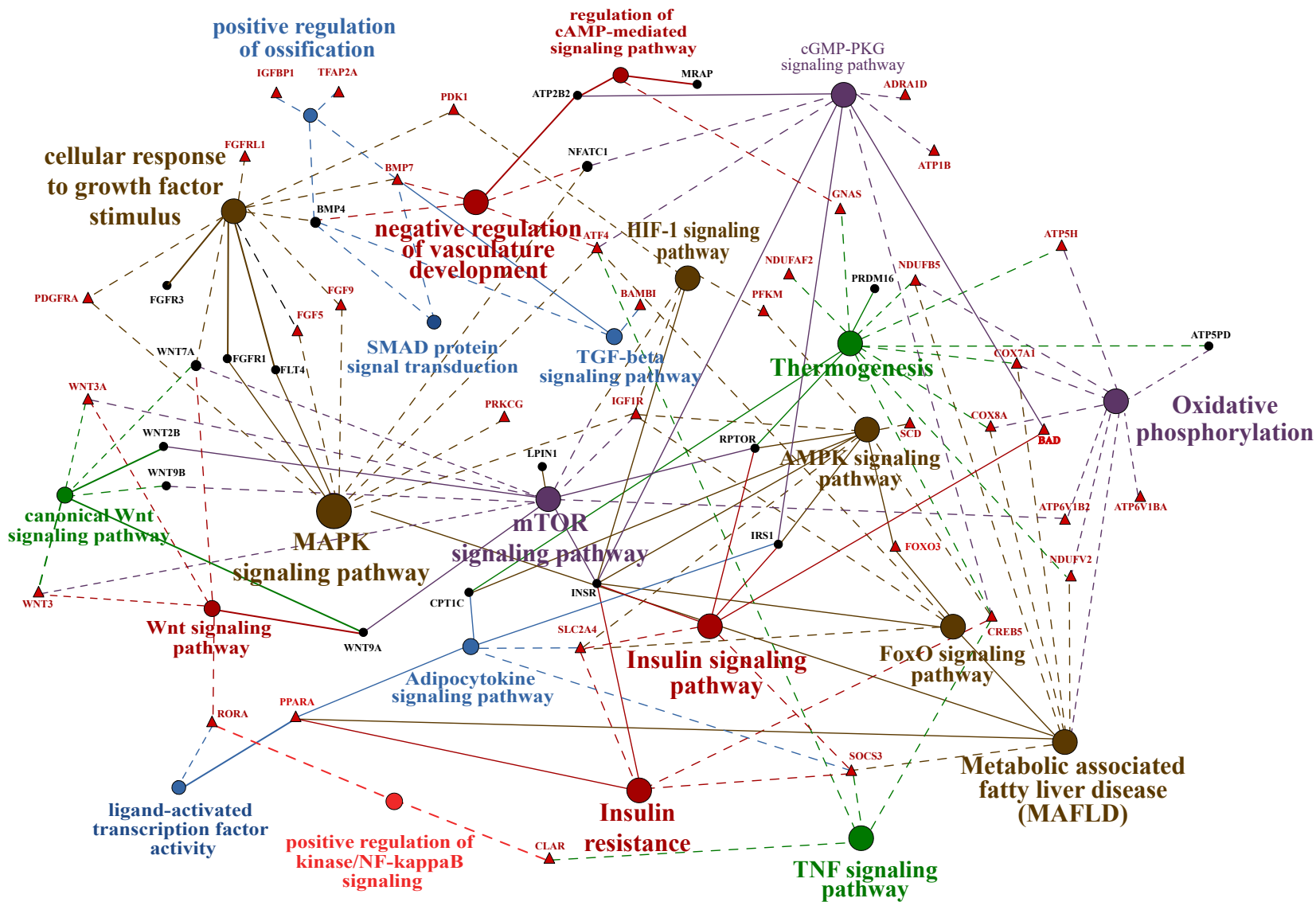

Supplement: Supplementary file 1 [file genes-12-00307-s001.zip › genes-1098573-supplementary/Figure S1.pdf]
